# A new generic circumscription of *Hydrochorea* (Leguminosae, Caesalpinioideae, mimosoid clade) with an amphi-Atlantic distribution

**DOI:** 10.3897/phytokeys.205.82775

**Published:** 2022-08-22

**Authors:** Marcos Vinicius Batista Soares, Erik Jozef Mathieu Koenen, João Ricardo Vieira Iganci, Marli Pires Morim

**Affiliations:** 1 Universidade Federal do Rio Grande do Sul, Programa de Pós-Graduação em Botânica, Av. Bento Gonçalves 9500, Bloco IV, Prédio 43433, 91501-970, Porto Alegre, Rio Grande do Sul, Brazil; 2 Rua dos Bandeirantes 1020, Caranazal, 68040-329, Santarém, Pará, Brazil; 3 Evolutionary Biology & Ecology, Université Libre de Bruxelles, Faculté des Sciences, Campus du Solbosch - CP 160/12, Avenue F.D. Roosevelt 50, 1050 Bruxelles, Belgium; 4 Instituto de Biologia, Universidade Federal de Pelotas, Campus Universitário Capão do Leão, Travessa Andre Dreyfus s/n, 96010-900, Capão do Leão, Rio Grande do Sul, Brazil; 5 Instituto de Pesquisas Jardim Botânico do Rio de Janeiro, Av. Pacheco Leão 915, 22460-030, Rio de Janeiro, Brazil

**Keywords:** *Albizia*, *Balizia*, *Cathormion*, Fabaceae, nomenclature, taxonomy

## Abstract

*Hydrochorea* and *Balizia* were established to accommodate four and three species, respectively, that were previously included in different ingoid genera, based primarily on differences in fruit morphology. Both genera have Amazonia as their centre of diversity, extending to Central America and the Brazilian Atlantic Rainforest. Previous phylogenetic evidence showed *Balizia* to be paraphyletic with respect to *Hydrochorea*, and species of *Hydrochorea* and *Balizia* were placed in a large unresolved polytomy with species of *Jupunba*. Here we present a new phylogenomic analysis based on 560 exons, from which 686 orthologous alignments were derived for gene tree inference. This analysis confirms a paraphyletic *Balizia* in relation to *Hydrochorea*, together with two African species formerly placed in *Albizia* nested within the clade. *Jupunbamacradenia* was resolved as sister to the clade combining those taxa. However, quartet support is low for several of the branches at the base of the clade combining the genera *Jupunba*, *Balizia* and *Hydrochorea*, suggesting that rapid initial divergence in this group led to extensive incomplete lineage sorting and consequently poor phylogenetic resolution. Because of these phylogenomic complexities, we decided to use morphology as the main guide to consider *Hydrochorea* as a distinct genus from *Jupunba*, and *Balizia* as a new synonym for *Hydrochorea*. The taxonomic treatment includes the study of collections from various herbaria and fieldwork expeditions. We present a re-circumscribed *Hydrochorea* accommodating a total of 10 species, including six new combinations, five new synonyms, one new taxonomic status, two corrections of nomenclature category for lectotypes, and a second step lectotype and three new lectotypes. A new species from the Brazilian Amazon is described and illustrated. An identification key for all species of *Hydrochorea* is presented, together with comments and illustrations.

## Introduction

Rupert C. Barneby and James W. Grimes established a new generic system for most of the ingoid mimosoids of the Americas in a landmark monographic series (Barneby and Grimes 1996, 1997; Barneby 1998). In it they created seven new genera, including *Hydrochorea* Barneby & J.W. Grimes and *Balizia* Barneby & J.W. Grimes, which were established to accommodate four and three species, respectively, that were previously included (as many ingoid species have been) in several different genera such as *Albizia* Durazz., *Arthrosamanea* Britton & Rose, *Cathormion* Hassk. and *Pithecellobium* Mart., among others (Lewis and Rico Arce 2005; Brown 2008). While Barneby and Grimes (1996: p. 35) stated that *Hydrochorea* and *Balizia* “have arisen from common ancestry”, these were nonetheless treated as separate genera based on differences in fruit morphology that were ascribed to adaptation to different habitats and seed dispersal strategies: *Hydrochorea* was defined based on lomentiform fruits adapted to water-borne dispersal in seasonally inundated habitats, while *Balizia* was described as a genus of “terra firme” forest (even though at least two of its three species were mentioned to also often occur on riverbanks), and recognized mainly based on having indehiscent or follicular fruits, with a septate endocarp but not lomentiform. Barneby and Grimes (1996) recognized *Balizia*, *Hydrochorea*, and *Abarema* Pittier s.l. as closely related genera, distinguished by fruit morphology (Iganci and Morim 2009, 2012). However, *Abarema* was shown to be polyphyletic (Iganci et al. 2016) and the type species of the genus, *Abaremacochliacarpos* (Gomes) Barneby & J.W. Grimes is placed in the Inga clade, together with the recently described *A.diamantina* E. Guerra, M.P. Morim & Iganci (Guerra et al. 2016, 2019). The other species of *Abarema* s.l. were segregated in the two genera *Jupunba* Britton & Rose and *Punjuba* Britton & Rose (Soares et al. 2021). These findings question all the former classifications of those taxa, which were mostly based on fruit morphology, and call for further studies aiming to better understand fruit and seed morphology in the context of the evolution of dispersal strategies and ecological adaptations.

Recent phylogenetic evidence (Iganci et al. 2016; Koenen et al. 2020b; Soares et al. 2021; Ringelberg et al. 2022; and a new analysis presented here) has shown *Balizia* to be paraphyletic with respect to *Hydrochorea*. Furthermore, two African species that were formerly placed in several genera including *Albizia* Durazz. and *Cathormion* Hassk. were also shown to be most closely related to *Hydrochorea* (Koenen et al. 2020b), and their general morphological features and ecology are virtually indistinguishable from Neotropical species of *Hydrochorea*.

Besides the advances in phylogenetic and phylogenomic methods, recent fieldwork collecting programmes have greatly contributed to herbarium collections of Amazonian taxa (Milliken et al. 2010; Cardoso et al. 2017; Ulloa et al. 2017; BFG 2021), and furthermore, the Reflora Program has led to the online availability of nearly all Brazilian plant collections (Pearce et al. 2020; BFG 2021), providing excellent opportunities for synoptic taxonomic revisions.

Here we present a taxonomic update including a new generic circumscription of *Hydrochorea* based on phylogenomic and morphological evidence, along with a nomenclatural review presented as a synopsis of the genus, which includes new combinations, new synonyms, and the description of a newly discovered species from the Upper Rio Negro. We include an identification key, illustrations, and distribution maps for the 10 species now accommodated in *Hydrochorea*.

## Materials and methods

### Taxonomy

Standard herbarium taxonomy practices were used for analysis of all species studied in the present work. The collections (including digital images) of the following herbaria were analysed: A, BM, BR, CTBS, E, F, G, GH, HUEFS, IAN, INPA, K, MG, MO, NY, P, PEL, R, RB, SP, US and Z (Thiers 2022). Fieldwork was carried out especially in the Upper Rio Negro region of Amazonian Brazil, where we collected four species of *Balizia* and *Hydrochorea*, including a species new to science. All the new collections were incorporated into the RB herbarium, in the Rio de Janeiro Botanical Garden, and duplicates were sent to partner institutions. Fresh leaf samples were stored in silica gel for total DNA extraction. We also visited the collections in NY to study the specimens that Barneby and Grimes (1996) worked with for their taxonomic account. Combined with studying the large number of new collections that have been made in the past 25 years since publication of the Barneby and Grimes (1996) taxonomic account, we were able to review their taxonomic decisions based on the relatively limited herbarium material available to them at the time.

Online databases were used to view digital images of specimens including types, especially the Reflora Virtual Herbarium (REFLORA -Herbário Virtual 2022), National Institute of Science and Technology (INCT-Splink 2022), and JSTOR (2022). Geographic distributions of each species were inferred based on specimen labels and literature (Barneby and Grimes 1996; Soares 2015). The morphological characters were described following Beentje (2016) and Iganci and Morim (2009, 2012), for habit, leaves, inflorescences, flowers, pods, and seeds. Original descriptions of all taxa were analysed, and nomenclature was revised according to the International Code of Nomenclature of algae, fungi and plants (the Shenzen code; Turland et al. 2018). Except for the new species described here, and the two African species now placed in *Hydrochorea*, all other species have been described earlier in detail by specialists (Barneby and Grimes 1996). Thus, for those species, here we only present new combinations, taxonomic notes and a reference to the literature where the complete description is available. Synonyms are accepted following Barneby and Grimes (1996) and are only listed here when either lectotypification or nomenclatural correction is needed.

### Exon selection, matrix assembly and phylogenomic analysis

To better evaluate the evidence for monophyly of the studied genera, or lack thereof, we have performed new analyses based on a selection of exons with flanking non-coding regions derived from the sequencing data of Koenen et al. (2020b) and Ringelberg et al. (2022), including network analyses and quantification of supporting bipartitions across gene trees for alternative topologies. The sequencing methods are described in those publications and here we only briefly describe our methods when they differ from Koenen et al. (2020b) and Ringelberg et al. (2022). In the original *Mimobaits* probe design (Koenen et al. 2020b), exons were predicted, and flanking untranslated regions (UTRs) were also (partially) included. From this reference exon set, we selected all of those that are longer than 500bp, which for initial or terminal exons includes the UTR. Read data of Koenen et al. (2020b) and Ringelberg et al. (2022) for the accessions of the Jupunba clade plus six outgroup accessions were mapped against these exons and non-matching reads discarded. Read quality filtering and *de novo* assembly methods followed Koenen et al. (2020b), and after clustering the assembled contigs to the reference sequences of the exons, initial alignments and gene trees were inferred using MAFFT (Katoh et al. 2005) and RAxML (Stamatakis 2014), respectively. Then, mono- and paraphyletic groups per species were collapsed to select a single allele in case multiple alleles were reconstructed (Yang and Smith 2014), followed by cutting long internal branches to splice potential paralogs into separate alignments using the cut_long_branches.py script of Yang and Smith (2014). The resulting clusters were then realigned with MAFFT and used to infer multilabeled gene trees using RAxML. Finally, gene trees with a single tip per species were extracted from these gene trees using the maximum inclusion (MI) method of Yang and Smith (2014) and used in species tree analysis in ASTRAL-III (Zhang et al. 2018) with default settings and nodes with less than 10% bootstrap support collapsed as suggested by the authors of the software. A filtered supernetwork was constructed in SplitsTree4 (Huson 1998) from the same set of gene trees but with nodes with less than 50% bootstrap support collapsed and with the mintrees parameter set to 100 (25% of the total number of gene trees). Quantification of supporting bipartitions across gene trees for alternative topologies followed the same methodology as Koenen et al. (2020a).

## Results

Our herbarium taxonomic work has resulted in the synopsis presented below. This includes a total of six new combinations, including a new status for a species that had been treated at varietal rank by Barneby and Grimes (1996), as well as the description of a new species. *Balizia* and its sections are placed in the synonymy of *Hydrochorea* and two new heterotypic synonyms, one at species and one at varietal rank, are proposed, as well as two lectotype corrections. A second step lectotype and three new lectotypes are designated.

### Phylogenomic analysis

**Figure 1.**
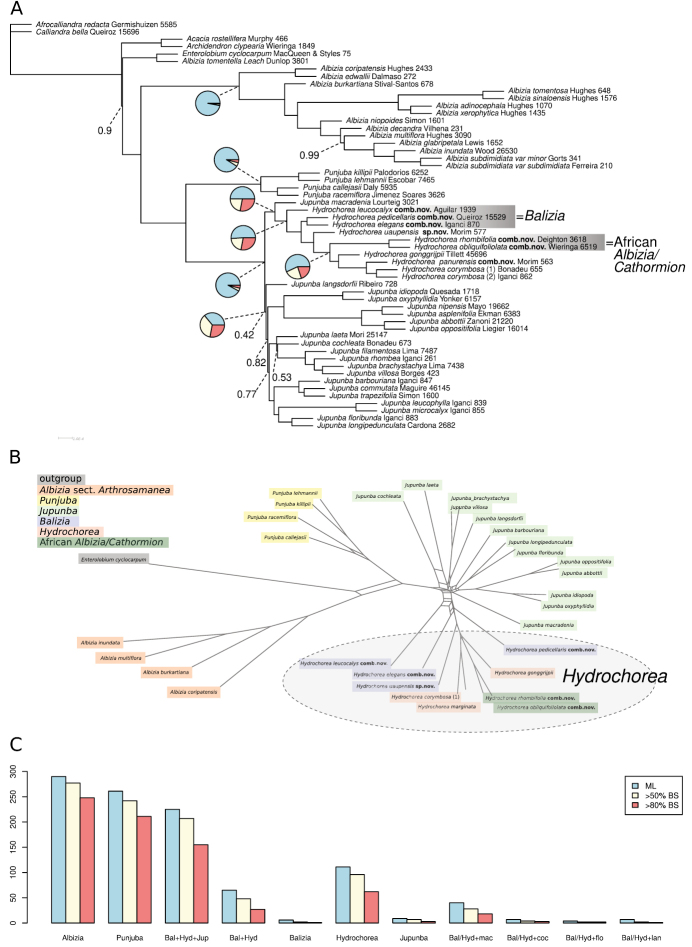
Phylogenomics of the Jupunba clade **A** ASTRAL-III species tree based on 398 gene trees, posterior probability values are shown only for those nodes for which support is lower than 1.0, and pie charts on several crucial nodes indicate alternative quartet support **B** filtered super-network of the same gene tree set with the genus *Hydrochorea* as circumscribed in this study indicated by a grey ellipse **C** bar graphs indicating numbers of compatible bipartitions across the same gene tree set in the maximum-likelihood estimate (ML) and when only taking into account bipartitions that receive at least 50 or 80% bootstrap support. The abbreviations that are used are Bal = *Balizia*, Hyd = *Hydrochorea*, and Jup = *Jupunba*. Note that the taxonomy of Albiziasect.Arthrosamanea is updated in this volume by Aviles Peraza et al. (2022), where new binomials in the genus *Pseudalbizzia* are presented for the majority of the species of this section.

A total of 560 exons were selected for gene tree inference, from which 398 MI gene trees were extracted after clustering and filtering (Suppl. materials 1, 2). The species tree based on these gene trees (Fig. 1A, Suppl. material 3) does not provide qualitatively different results from those of Ringelberg et al. (2022), showing a paraphyletic *Balizia* relative to *Hydrochorea* together with the two African *Cathormion*/*Albizia* species. All currently recognised species of *Balizia* and *Hydrochorea*, including the type species of both genera, were sampled, with the exception of *H.marginata*, although this species was included in the studies of Iganci et al. (2016) and Soares et al. (2021). Also, *Jupunbamacradenia* (Pittier) M.V.B. Soares, M.P. Morim & Iganci was found as sister of the clade combining these genera. However, quartet support is low for several of the branches at the base of the clade combining the genera *Jupunba*, *Balizia* and *Hydrochorea*, suggesting that rapid initial divergence in this group led to extensive incomplete lineage sorting and consequently poor phylogenetic resolution. This is further reinforced by the filtered supernetwork (Fig. 1B), which also clearly shows the paraphyly of *Balizia*, and shows that *Hydrochorea* and *Balizia* together form a group that is separate from *Jupunba*, but with a complex network structure indicative of incomplete lineage sorting. When the monophyly of these genera and their sister-group relationships are evaluated based on the number of compatible bipartitions across gene trees, it is clear that Albiziasect.Arthrosamanea (Britton & Rose) Barneby & J.W. Grimes, *Punjuba* and *Hydrochorea* (including the two African *Cathormion*/*Albizia* species) are each monophyletic, while *Balizia* and *Jupunba* are only supported to be monophyletic by a small number of gene trees (Fig. 1C). Support for the sister-group relationship of *Jupunbamacradenia* with *Balizia* + *Hydrochorea* is supported by c. 10% of the gene trees. This is higher than what is found for some other species of *Jupunba* that are sister to *Balizia* + *Hydrochorea* in some gene trees, but nonetheless the separation between these genera is not very clear likely due to significant incomplete lineage sorting.

## Discussion

In this study, we have made an in-depth investigation of the generic delimitation issues surrounding the genera *Balizia*, *Cathormion* and *Hydrochorea*, to reconcile morphological characters of the group with phylogenetic relationships and to propose a revised classification. While the uncovered phylogenomic complexity adds further difficulty to the goal of achieving a stable classification for these taxa, we conclude that the taxa with either indehiscent, follicular or lomentiform fruits, that are septate between the seeds at least in the endocarp, are preferably all classified within a recircumscribed *Hydrochorea*, separate from the genus *Jupunba* which is characterized by dehiscent fruits that are never septate between the seeds. Extensive incomplete lineage sorting surrounding the early evolution of these genera means that they are phylogenomically not well separated (Fig. 1), but in order to ensure both diagnosability and stability of names, we believe keeping these as separate genera is justified (i.e., transferring some species to *Hydrochorea* to account for the non-monophyly of *Balizia* is preferable to moving all taxa to a morphologically heterogeneous *Jupunba*).

One of the most interesting aspects of *Hydrochorea* is the evolution of its fruit morphology and dehiscence in adaptation to water-borne seed dispersal, which presumably led to its distribution in riparian, swamp and periodically inundated forests on both sides of the Atlantic, as trans-oceanic dispersal is presumed to be relatively likely in hydrochorous plants. Much attention was traditionally given to pod morphology in mimosoids, in attempts to classify the ingoid genera, as one of the most easily observable characters to visually distinguish the taxa (Barneby and Grimes 1996; Lewis and Rico-Arce 2005). Lomentiform pods are typical of most species of *Hydrochorea*, but are also found in *Albizia* s.s. (*Albiziadolichadena* I.C. Nielsen, *Albiziamoniliformis* (DC.) F. Muell., *Albiziarosulata* (Kosterm.) I.C. Nielsen and *Albiziaumbellata* (Vahl) E.J.M. Koenen) and Albiziasect.Arthrosamanea (Barneby and Grimes 1996; Aviles Peraza et al. 2022). The craspedia, as found in *Mimosa* L., *Adenopodia* C. Presl and *Entada* Adans., are somewhat similar, but in those genera there is a replum (a persistent framework formed by the upper and lower suture of the craspedium) that stays attached to the infructescence after the 1-seeded articles have been shed. The fruit of *Cathormionaltissimum* (Hook. f.) Hutch. & Dandy (which is transferred to a new genus by Koenen 2022) is also similar, but the fruit of that species differs in containing aerenchymous tissue on the seminiferous nuclei to promote floating. These various lomentiform ingoid fruits also differ in whether they break up while still attached to the tree as is the case in most species of *Hydrochorea* and some species of Albiziasect.Arthrosamanea, or whether the fruit falls from the tree entire and only tardily breaks up into 1-multiple seeded articles afterwards, as appears to be the case in most of the other genera. Phylogenetic evidence clearly indicates that these fruits have all evolved independently from one another (Ringelberg et al. 2022), presumably in response to adaptation to riparian and periodically inundated habitats. The homoplasious nature of these similar fruits has led to many species having been moved around between different genera, based on their fruit morphology (Nielsen 1981). We note that the legume fruit appears to be amenable to the evolution of lomentiform fragmentation, given the similar craspedia that were independently derived at least twice and, moreover, lomentiform fruits are also present in several lineages of subfamily Papilionoideae.

Recent advances in molecular systematics of ingoid legumes also demonstrated the pod morphology to be less informative than previously thought (Souza et al. 2013; Iganci et al. 2016; Koenen et al. 2020b; Soares et al. 2021; Souza et al. 2022). Barneby and Grimes (1996) stated that *Balizia* and *Hydrochorea* closely resemble *Albizia* but are distinguished by indeterminate inflorescence-axes and vegetative branches arising from sylleptic and proleptic buds, pinnate leaflet venation, and truncate ovaries. The authors also highlighted the strong similarities in flower morphology shared between *Abarema* s.l., *Balizia* and *Hydrochorea*.

Iganci et al. (2016) found the species of *Hydrochorea* and *Balizia* in a large unresolved polytomy together with *Jupunba* species. The Bayesian results of Soares et al. (2021) are in line with the generic delimitation proposed here, with 1.0 posterior probability supporting *Jupunba*, *Hydrochorea* and *Balizia* as monophyletic considering matK sequences only, and a monophyletic *Jupunba* and paraphyletic *Hydrochorea* in relation to *Balizia* when considering ETS sequences only. However, there was no bootstrap support for a monophyletic *Jupunba* and neither for the clade uniting *Hydrochorea* and *Balizia*. The results of Ringelberg et al. (2022) show that *J.macradenia* is more closely related to *Hydrochorea* and *Balizia* than to other species of *Jupunba*, in contrast to the phylogenetic position of the same accession in Soares et al. (2021), but in accordance with the phylogenomic results presented here. Notably, Soares et al. (2021) included three accessions of *J.macradenia*, which were firmly nested in a monophyletic *Jupunba*, suggesting that further phylogenomic analyses with more accessions included will need to be carried out to further test the monophyly of *Jupunba*.

Phylogenetic evidence (Iganci et al. 2016; Koenen et al. 2020b; Soares et al. 2021; Ringelberg et al. 2022; and a new analysis presented here) that shows *Balizia* to be paraphyletic with respect to *Hydrochorea*, as well as the field discovery of a new species that is morphologically intermediate between the two genera, with crypto-lomentiform pods that resemble more the follicle of *Baliziapedicellaris* (DC.) Barneby & J.W. Grimes than the lomentiform pods of *Hydrochorea*, but a species of seasonally inundated forest with hydrochorous seed dispersal, prompted us to decide that the two genera are best combined. Furthermore, two African species that were formerly placed in several genera, including *Albizia* and *Cathormion*, were shown to be closely related to *Hydrochorea* based on phylogenomic evidence (Koenen et al. 2020b). These species are therefore best accommodated within *Hydrochorea*, as their general morphological features and ecology are virtually indistinguishable from Neotropical species of *Hydrochorea*.

Koenen et al. (2020b) and Ringelberg et al. (2022) also show that Albiziasect.Arthrosamanea is placed in the Jupunba clade, being more closely related to *Jupunba*, *Punjuba*, *Balizia* and *Hydrochorea* than to *Albizia* s.s. These results are reinforced by Aviles Peraza et al. (2022) who proposed nomenclatural updates to solve this situation based on more extensive sampling of Albiziasect.Arthrosamanea. In our study (Fig. 1), we have still referred to these species as Albiziasect.Arthrosamanea but we refer the reader to Aviles Peraza et al. (2022) for presentation of a new taxonomy of the group.

Based on parsimony analysis of morphological characters only, Barneby and Grimes (1996) recognized pollen polyads comprising 16 grains as a synapomorphy for the Abarema alliance, while other alliances that they recognized (e.g., the Samanea, Chloroleucon and Inga alliances) presented a polymorphic polyad number. Indeed, Guinet and Grimes (1997) studied all Neotropical species of *Hydrochorea* and *Balizia* for their polyads and we observed that the nested African species *Cathormionobliquifoliolatum* (De Wild.) G.C.C. Gilbert & Boutique (*Deighton 3618*, K) and *Cathormionrhombifolium* (Benth.) Keay (Germain 87, K) also have 16-celled polyads. Polyad morphology was also highlighted as diagnostic for the recognition of *Afrocalliandra* E.R. Souza & L.P. Queiroz, a genus segregated from *Calliandra* Benth. (Souza et al. 2013), and more attention should be given to this character in future studies. Furthermore, the taxa in the Jupunba clade share the simultaneous presence of vegetative and reproductive branches (sylleptic), a character considered by Grimes (1999) as uncommon amongst the ingoid legumes. Thus, possessing polyads with 16 grains and sylleptic branches could circumscribe either the clade comprised of *Hydrochorea*, *Jupunba* and *Punjuba*, or a more conservative *Jupunba* s.l. Forthcoming studies, including more samples of unstable taxa in current molecular analyses, new field collections and advances in phylogenomic analysis, will hopefully resolve this question.

We did not include *Hydrochoreaacreana* (J.F. Macbr.) Barneby & J.W. Grimes in our synopsis and the name is here considered as *incertae sedis*. Pods from this species were not known to Barneby and Grimes (1996), and herbarium specimens are difficult to identify when flowers and especially fruits are unavailable. The type specimen (*Krukoff 5631*, NY334624) includes flowers arranged in a large terminal panicle composed of umbelliform pseudoracemes of capitula, differing from the axillary to terminal inflorescences in *Hydrochorea* and *Jupunba* that are not paniculate, and always have sylleptic branches present. Citing fruiting Central American collections, *Hydrochoreaacreana* was combined into *Abarema* s.l. by Rico-Arce (1999) as *Abaremaacreana* (J.F. Macbr.) L. Rico. However, the Central American collections cited by Rico-Arce (1999) were identified by Barneby and Grimes as either *Abaremamacradenia* (Pittier) Barneby & J.W. Grimes or *Abaremaadenophora* (Ducke) Barneby & J.W. Grimes, with which we agree. Interestingly, a specimen from Acre which was collected in 1995 (*Oliveira 691*; NY00662831) and was presumably not seen by Barneby and Grimes (1996), but was identified as *A.acreana* by L. Rico-Arce, does include unripe pods and this material is likely conspecific with the type material of *H.acreana*. However, as discussed before, fruit morphology often has been shown to be rather misleading in mimosoid taxonomy due to homoplasy, and we point out the differences in inflorescence structure from *Jupunba* as discussed above. Soares et al. (2021) resolved *H.acreana* as sister to *Albiziasubdimidiata* (Splitg.) Barneby & J.W. Grimes, and not closely related to *Hydrochorea* nor *Jupunba*, but these analyses were based on ETS sequences only and morphologically the material does not bear much resemblance to species of Albiziasect.Arthrosamanea, in which *Albiziasubdimidiata* is placed. Given the taxon’s unusual combination of morphological characters, it may well represent an isolated lineage that merits recognition as a distinct genus; this decision is pending further study.

### Taxonomic treatment

#### Hydrochorea

Taxon classificationPlantaeFabalesFabaceae

Barneby & J.W. Grimes, Mem. New York Bot. Gard. 74(1): 23. 1996.

EBB162E5-9FC3-52D9-A745-335989AF21FE

Figs 2 , 3

Balizia Barneby & J.W. Grimes, syn. nov., Mem. New York Bot. Gard. 34(1). 23. 1996. Type: Baliziapedicellaris (DC.) Barneby & J.W. Grimes.
Balizia sect. Leucosamanea Barneby & J.W. Grimes, syn. nov., Mem. New York Bot. Gard. 34(1). 36. 1996. Type: Balizialeucocalyx (Britton & Rose) Barneby & J.W. Grimes.
Balizia Barneby & J.W. Grimes sect. Balizia syn. nov., Mem. New York Bot. Gard. 34 (1). 37. 1996. Type: Baliziapedicellaris (DC.) Barneby & J.W. Grimes.

##### Type.

*Hydrochoreacorymbosa* (Rich.) Barneby & J.W. Grimes.

##### Description.

**Shrubs** and **trees**, unarmed; branches grey to brown pilosulous to glabrescent, cylindrical; stipules persistent or caducous. **Leaves** bipinnate, with 1–15 pairs of pinnae; petiole canaliculate or cylindrical, grey to brown pilosulous or glabrous; nectaries sessile to stipitate, orbicular, patelliform, or cupuliform, the first either near mid-petiole or between the first pinnae pair, and often along the leaf rachis, between the leaflet pairs; leaflets 2–33 pairs per pinna, petiolate to subsessile, rhombic-ovate, rhombic-lanceolate, rhombic-oblong, rhombic-obovate, ovate, elliptic, oblong, lanceolate or oblanceolate, grey to brown pilosulous, ciliate or glabrous, concolorous or more often discolorous, venation pinnate. **Inflorescence** consisting of umbelliform capitula or corymbiform racemes, arising singly or fasciculate from the axils of coeval or hysteranthous leaves, bracts generally caducous; bracteoles persistent or caducous. **Flowers** heteromorphic, pedicellate in peripheral flowers, mostly pentamerous, and sessile in the larger terminal flower, 5–8-merous; calyx green, gamosepalous, campanulate, or tubular, pubescent, ciliate or glabrous; corolla pinkish to reddish, yellowish or whitish, gamopetalous, infundibuliform, campanulate, or tubular, glabrous, puberulent, ciliate or pilose at the apex; androecium with (10–)12–60(–75) stamens; filaments white, greenish or roseate, fused into a tube, included in peripheral flowers or exserted beyond the corolla in the terminal flower; stemonozone present, anthers dorsifixed; ovary superior, sessile, truncate at the apex, usually pubescent or sometimes glabrous. **Fruits** sessile or shortly stipitate, straight or slightly recurved, either lomentiform, the seeds released in one-seeded articles, or woody and indehiscent, the exocarp with transverse fibres and the endocarp hard and septate, or follicular, with similar exocarp but the septate endocarp papyraceous and shed along with the seeds, or crypto-lomentiform with follicular dehiscence, the exocarp smooth and the endocarp remaining attached to the seeds forming 1-seeded articles. **Seeds** with a hard testa, with pleurogram complete or narrowly U-shaped.

##### Distribution and habitat.

**Figure 2.**
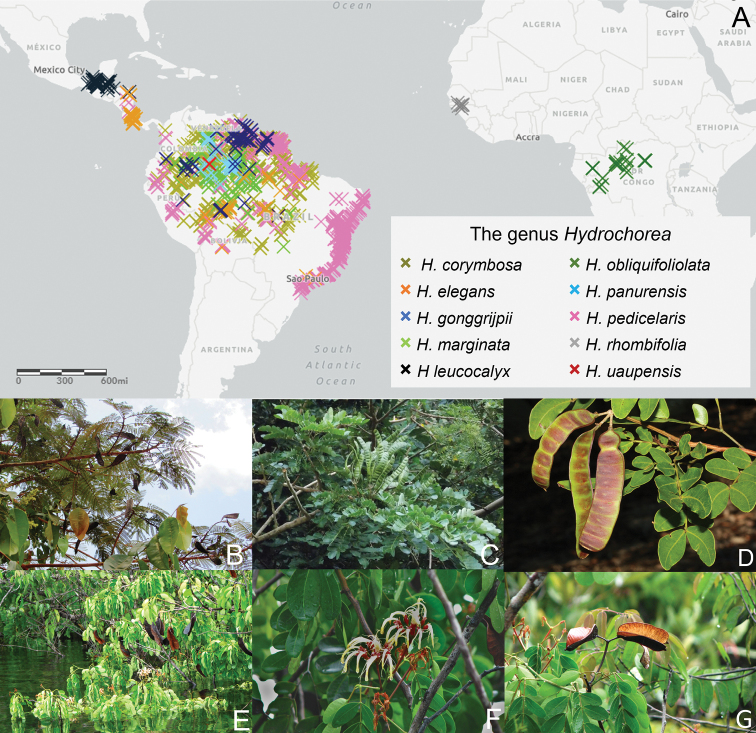
The genus *Hydrochorea* Barneby & J.W. Grimes **A** The amphi-atlantic geographic distribution of *Hydrochorea***B***Hydrochoreapedicellaris* (DC.) M.V.B. Soares, Iganci & M.P. Morim foliage and fruits **C***Hydrochoreacorymbosa* (Rich.) Barneby & J.W. Grimes foliage and fruits **D***Hydrochoreapanurensis* (Spruce ex Benth.) M.V.B. Soares, M.P. Morim & Iganci foliage and fruits **E***Hydrochoreauaupensis* M.P. Morim, Iganci & E.J.M. Koenen in habitat, with foliage and fruits **F** Flowers of *H.uaupensis* after rain **G** mature fruits of *H.uaupensis*. **B, C** from M.V.B Soares **D** from D. Cardoso **E–G** from J.R.V. Iganci.

North America (Mexico), Central America (Belize, Costa Rica, Guatemala, Honduras and Nicaragua), South America (Brazil, Bolivia, Colombia, Ecuador, French Guiana, Guyana, Peru, Suriname and Venezuela) and Africa (Congo Basin and West Africa) (Fig. 2A). *Hydrochorea* species occur in riparian habitats, inundated and non-inundated wet tropical forests of the Orinoco and Amazon basins, pre-Andean Amazonia along the Nor-Yungas and Pando in Bolivia, Vaupés in Colombia and Huánuco in Peru, Central Brazilian Savanna, the Atlantic Rainforest of Brazil and extending to northern South America in Venezuela and the Guianas and the Gulf-Caribbean lowlands until Mexico, and one species in coastal tidal swamp forests in Upper Guinea (West Africa) and one species in riparian and seasonally inundated forests in the Congo Basin.

##### Note.

Since the names *Hydrochorea* and *Balizia* were published in the same publication (Barneby and Grimes 1996), neither has priority, although *Hydrochorea* was treated as genus 1 and *Balizia* as genus 2, *Hydrochorea* thus appearing first in the publication. The name *Hydrochorea* is here chosen to represent the recircumscribed genus, especially since the name is appropriate for most of its species, and most *Balizia* species are also thought to frequently use water-borne seed dispersal, and all but one species (*B.elegans*) are reported to often occur along river-banks. The name *Balizia*, being an anagram of *Albizia*, is less appropriate given that several of its species have previously been placed in *Albizia* and therefore the name may suggest close kinship, while actually being most closely related to the genus *Jupunba*.

### Identification key to the species of *Hydrochorea*

**Table:** 

1a	Species from Congo Basin and West Africa	**2**
2a	Adaxial leaflet surface shiny, abaxial leaflet surface glabrous, apart from the ciliate midrib or with few scattered short hairs especially on and near the midrib; calyx and corolla green to greenish white, corolla lobes glabrous or with a few short white hairs around the apex, Congo Basin (Democratic Republic of Congo, Central African Republic and Gabon)	**6. *H.obliquifoliolata***
2b	Adaxial leaflet surface dull, abaxial leaflet surface pilose with varying density of hairs (rarely nearly glabrous); calyx and corolla white, upper half of corolla lobes rusty pilose to villous, West Africa (Senegal, Guinea-Bissau, Guinea, and Sierra Leone)	**9. *H.rhombifolia***
1b	Species from North, Central and South America	**3**
3a	Pinnae 1–jugate on every leaf (seldom 2-jugate and then the true petiole very short)	**4**
4a	Calyx covering the corolla in bud; flowers glabrous, terminal flower with tubular calyx	**7. *H.panurensis***
4b	Calyx not covering the corolla in bud; flowers puberulous, terminal flower with campanulate calyx	**5. *H.marginata***
3b	Pinnae 2– or more jugate (seldom 1–jugate on some leaves of the same individual)	**5**
5a	Leaflets up to 10 pairs per pinna	**6**
6a	Pinnae 1–2 jugate, leaflets ovate to rhombic-ovate, corolla of peripheral flowers up to 1.5 mm long; follicular crypto-lomentiform fruit	**10. *H.uaupensis***
6b	Pinnae (2–)3–6-jugate; leaflets rhombic-oblong, rhombic-ovate or rhombic-lanceolate; corolla of peripheral flowers with more 1.5 mm long; fruit indehiscent or lomentiform	**7**
7a	Leaflets rhombic-oblong; corolla of peripheral flowers more than 7 mm long	**4. *H.leucocalyx***
7b	Leaflets rhombic-ovate to rhombic-lanceolate; corolla of peripheral flowers up to 6 mm long	**1. *H.corymbosa***
5b	Leaflets in more than 10 pairs per pinna	8
8a	Corolla of peripheral flowers 8–10 mm long, fruit indehiscent, not lomentiform	**2. *H.elegans***
8b	Corolla of peripheral flowers up to 7.5 mm long, fruit lomentiform or follicular	**9**
9a	Pinnae 3–5-jugate; fruit lomentiform	**3. *H.gonggrijpii***
9b	Pinnae 6–17-jugate; fruit follicular, with septate endocarp and transverse fibers in the exocarp	**8. *H.pedicellaris***

#### 1. Hydrochorea corymbosa

Taxon classificationPlantaeFabalesFabaceae

(Rich.) Barneby & J.W. Grimes, New York Bot. Gard. 74(1): 27. 1996.

DAD0E817-D749-5B44-A35A-CA0660B3173F

Fig. 2A, C , 3A-C , 4

Pithecellobium subcorymbosum Hoehne [as Pithecolobium], Comiss. Linhas Telegr. Estratég., Mato Grosso-Amazonas, Bot. 8: 18, Ic. 133. 1919. Type: Brazil, Mato Grosso, São Luiz de Cáceres, nas margens do rio Paraguai, perto da Campina, *Hoehne 4582* (lectotype, designated here from amongst the syntypes: R! [R000003169]; isolectotype: SP).

##### Basionym.

*Mimosacorymbosa* Rich., Actes Soc. d’Hist. Nat. Paris 1: 113. 1792.

**Figure 3.**
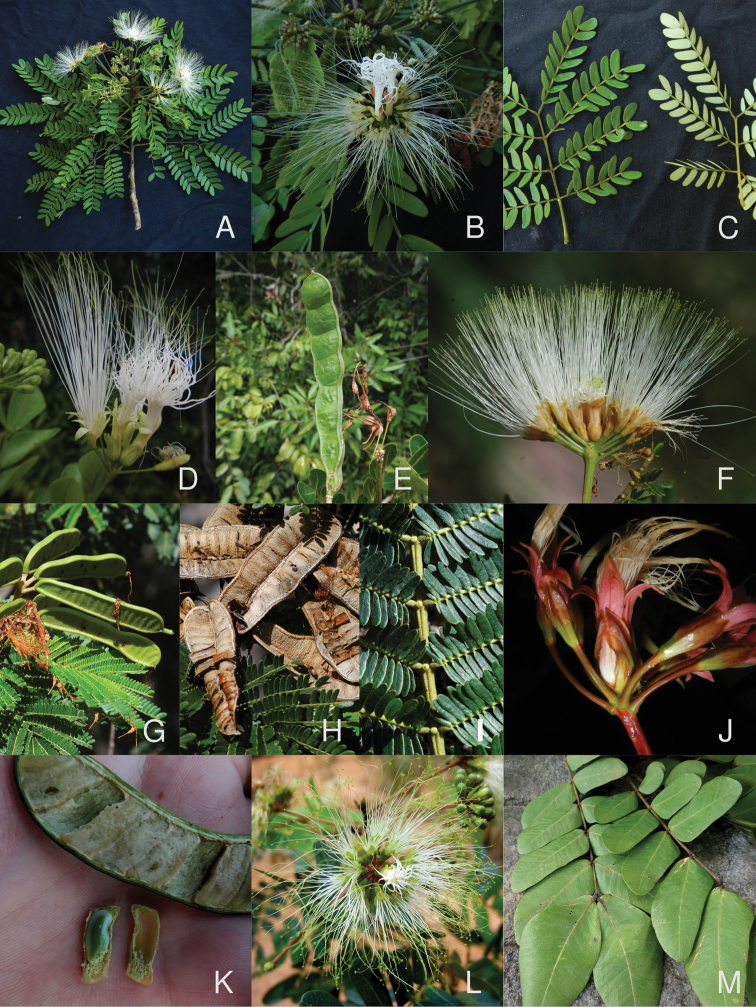
The genus *Hydrochorea* Barneby & J.W. Grimes (continued). Species from the Americas **A** flowering branch of *Hydrochoreacorymbosa* (Rich.) Barneby & J.W. Grimes **B** close-up of inflorescence of *H.corymbosa***C** discolorous leaves of *H.corymbosa***D** close-up of inflorescence of *Hydrochoreapanurensis* (Spruce ex Benth.) M.V.B. Soares, M.P. Morim & Iganci **E** unripe lomentiform pod of *H.panurensis***F** close-up of inflorescence of *Hydrochoreapedicellaris* (DC.) M.V.B. Soares, Iganci & M.P. Morim, with a few peripheral flowers removed to expose sessile terminal flowers **G** unripe pods of *H.pedicellaris***H** dehisced follicular pods of *H.pedicellaris* showing papery septate endocarp **I** detail of primary rachis of *H.pedicellaris* showing interpinnal extra-floral nectaries **J** inflorescence of *Hydrochoreauaupensis* M.P. Morim, Iganci & E.J.M. Koenen showing large sessile central flower and pedicellate peripheral flowers **K** unripe crypto-lomentiform pod and seed enveloped by septate endocarp of *H.uaupensis*; African species **L** inflorescence of *Hydrochoreaobliquifoliolata* (De Wild.) E.J.M. Koenen **M** pinnae of *Hydrochorearhombifolia* (Benth.) E.J.M. Koenen showing rhombic leaflets. **A–E, J, K** Erik Koenen **F-I** Colin Hughes **L** Jan Wieringa **M** William Hawthorne. Vouchers **A–C***J.R.V. Iganci 862***D, E***M.P. Morim 563***F–I***L.P. Queiroz 15529***J, K***M.P. Morim 577***L***J.J. Wieringa 6519***M** unvouchered.

**Figure 4.**
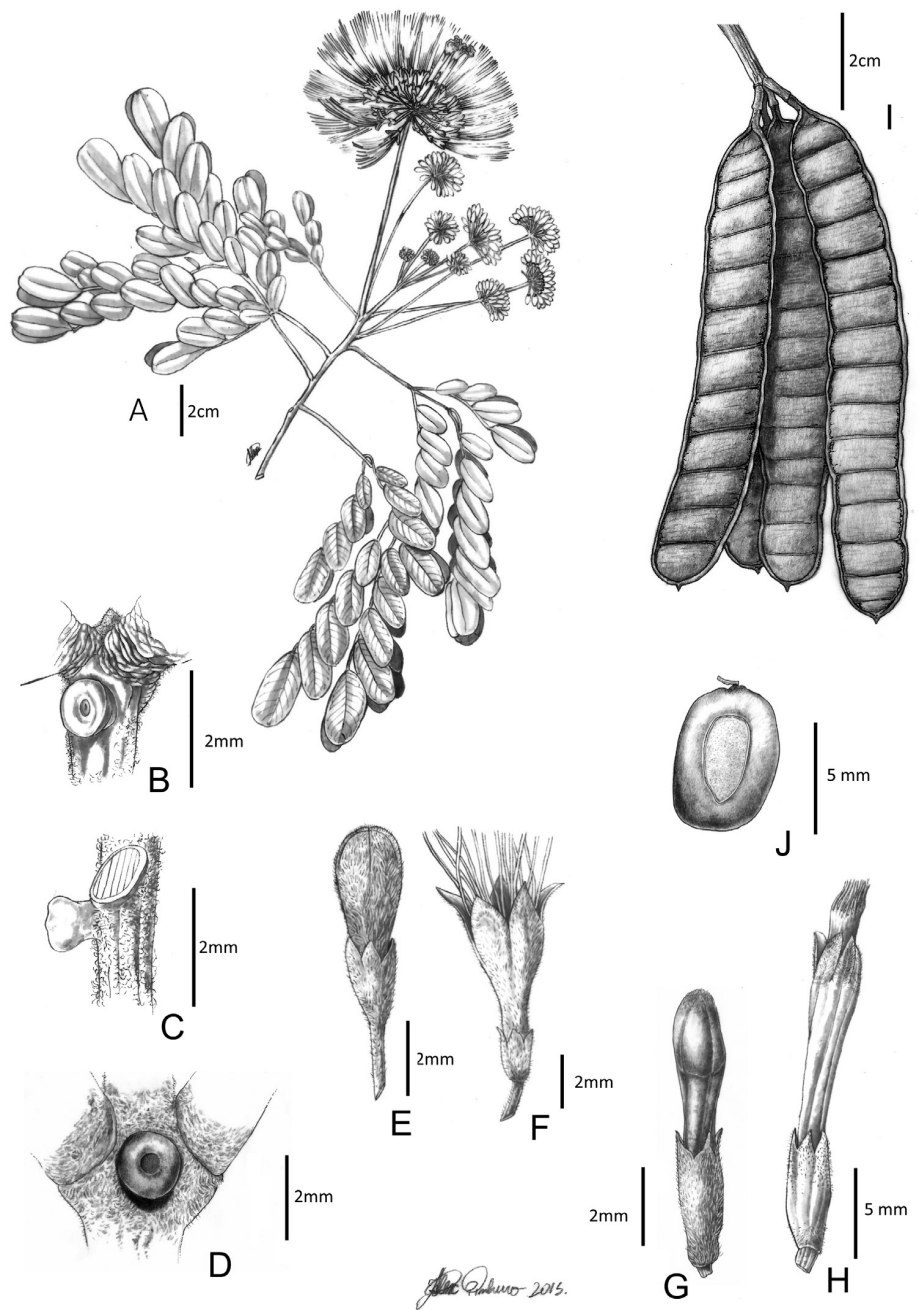
*Hydrochoreacorymbosa* (Rich.) Barneby & J.W. Grimes **A** branch with inflorescences **B–D** extra-floral nectaries **E** peripheral flower bud **F** peripheral flower **G** terminal flower bud **H** terminal flower **I** fruit **J** seed. **A, E–H** from *M.V.B. Soares 75***B–D** from *M.V.B. Soares 180***I–J** from *M.V.B. Soares 174*. Illustration by Alex Pinheiro.

##### Type material.

**French Guiana**, frequens in sylvis ripariis fluvii Kourou, *Louis Claude Richard s.n.* (lectotype, designated by Barneby and Grimes 1996, p. 27, as holotype, here corrected: P [P02142909] digital image!).

##### Distribution and habitat.

Bolivia, Brazil, Colombia, Ecuador, French Guiana, Guyana, Paraguay, Peru, Venezuela. *Hydrochoreacorymbosa* occurs in periodically or permanently inundated riparian forest, gallery forest, and open vegetation, up to 480 m elevation (Barneby and Grimes 1996).

##### Notes.

*Hydrochoreacorymbosa* is morphologically similar to *H.gonggrijpii* by its leaves with (2–)3–6 pairs of pinnae (3–5 pairs of pinnae in *H.gonggrijpii*), but differs by presenting (4–)5–11(–14) leaflet pairs per pinnae (vs. (12–)14–35 in *H.gonggrijpii*). *Hydrochoreacorymbosa* has a wide distribution in the Brazilian Amazon, and displays wide morphological plasticity. Barneby and Grimes (1996) recognised the specimen *Louis Claude Richard s.n.* (P02142909) as holotype, although the species protologue did not present a type specimen. The specimen does represent original material that the author associated with the taxon, being a specimen collected by the author and annotated as *Mimosacorymbosa*. Thus, *Louis Claude Richard s.n.* (P02142909) is here corrected to lectotype (Art. 9.3, 9.4, 9.8 and 9.10; Turland et al. 2018).

##### Selected specimens examined.

Brazil, Amazonas: São Gabriel da Cachoeira, entre Assunção do Içana e Camarão, mato de Igapó, margem do rio, 10 July 2012, *J.R.V. Iganci 862* (RB). Bolivia, Pando: Federico Roman, bordo del Río Abuna, 18 November 2006, *S. Altamirano & H. Ramos 4293* (K). Colombia, Vaupés: Mitú and Vicinity, lower rio Kubiyú, 26 September 1976, *Zarucchi 2147* (INPA). Ecuador, Francisco de Orellana: Estación Científica Yasuní, Río Tiputini, este de la Carretera Repsol-YPF, km 7 desvío hacia el pozo Tivacuno, Laguna Herradura, 20 April 1999, *G. Villa 177* (K). Guyana: Potaro-Siparuni, riparian zone lower Kuribrong, April 2010, *Zartman et al. 8002* (INPA). Peru, Loreto: Jenaro Herrena, Cano Supay, flooded forest along cano, 23 May 2002, *T.D. Pennington et al. 17430* (K). Venezuela, Amazonas: Departamento Rio Negro, middle part of the Río Baria, 21 July 1984, *G. Davidse 27570* (K).

#### 2. Hydrochorea elegans

Taxon classificationPlantaeFabalesFabaceae

(Ducke) M.V.B. Soares, Iganci & M.P. Morim comb. nov.

C88905FF-CD0D-5E1C-8F7A-C41EA9C07A4E

urn:lsid:ipni.org:names:77303827-1

Fig. 2A

Balizia elegans (Ducke) Barneby & J.W. Grimes, Mem. New York Bot. Gard. 74(1): 40 1996. Albiziaelegans (Ducke) L. Rico, Novon, 9(4): 556. 1999.
Albizia duckeana L. Rico, syn. nov., Kew Bull. 55(2): 404. 2000. Type: based on Pithecellobiumelegans Ducke.

##### Basionym.

*Pithecellobiumelegans* Ducke [as *Pithecolobium*], Arch. Jard. Bot. Rio de Janeiro 3: 64. 1922.

##### Type material.

Brazil, in silvis non inundatis, prope Alcobaca (Tocantins), *A. Ducke 16271* (lectotype, designated by Barneby and Grimes 1996, p. 40: MG [MG00016271], digital image!; isolectotypes: G [G00359898] digital image!, MG, P [P03093819] digital image!, R [R000002384] digital image!, RB [RB10177]!, US [US1040853] digital image!, US [US00000336] digital image!, US [US00610722] digital image!).

##### Distribution and habitat.

Bolivia, Brazil, Costa Rica, Ecuador, Honduras, Nicaragua, Peru. *Hydrochoreaelegans* occurs in primary rain forest, up to 350 m elevation (Barneby and Grimes 1996).

##### Notes.

*Hydrochoreaelegans* has a morphological affinity with *H.pedicellaris*, as already pointed out by Ducke (1922) and by Barneby and Grimes (1996). However, the corolla of peripheral flowers is larger (8–10 mm long) in *H.elegans* than in *H.pedicellaris* (up to 7.5 mm long). Ducke (1922) and Barneby and Grimes (1996) also commented on the similarity between the fruit of both species, but the fruits of *H.elegans* are indehiscent (vs. follicular dehiscence in *H.pedicellaris*). *Hydrochoreaelegans* has a disjunct distribution between hylaean Brazil and Costa Rica and Nicaragua.

##### Selected specimens examined.

Brazil, Rondônia: Porto Velho, área do Reservatório da Usina Hidrelétrica de Samuel, 15 June 1986, *C.A.C. Ferreira 7458* (K). Costa Rica, Limón: Talamanca, Fila Carbon, Finca de Pedro Bolivar, 25 May 1999, *O. Valerde 1175* (K).

#### 3. Hydrochorea gonggrijpii

Taxon classificationPlantaeFabalesFabaceae

(Kleinhoonte) Barneby & J.W. Grimes, Mem. New York Bot. Gard. 74 (1): 25. 1996.

24CE013B-BE4C-5C34-9CDD-75A09762713F

Figs 2A , 5

##### Basionym.

*Pithecellobium* [as *Pithecolobium*] *gonggrijpii* Kleinhoonte Recueil Trav. Bot. Néerl. 22: 414. 1926.

##### Type material.

Suriname, im Reservat der Zanderij I, die nummerierten Baume n. 102 (Herb. [Acad.Rhenotraiect.J n. 1529, im Dez. 1915, und n. 4350bl. im Juli 1919) und n. 141 (Herb. n. 4357, bl. Im Juli 1919.)” 141, 10/VII/1919”, *Forest Bureau 4357* (lectotype, designated here from amongst the syntypes: IAN [IAN49436]!; isolectotypes: A [A00064017] digital image!, BR [BR0000005170067] digital image!, K [K000527996]!, K [K000527995]!, MO [MO954361] digital image!, NY [NY00334660] digital image!, NY [NY00334661] digital image!, NY [NY00334662] digital image!, P [P01818508] digital image!, U [U U0003385] digital image!, U [U0003384] digital image!, US [US00629380] digital image!).

##### Distribution and habitat.

Brazil, Colombia, Guyana, French Guiana, Suriname, Venezuela. *Hydrochoreagonggrijpii* occurs along riverbanks, gallery forest margins, and low-lying swamp forests, at 40–1400 m elevation (Barneby and Grimes 1996).

##### Notes.

In the nomenclatural treatment of *H.gonggrijpii*Barneby and Grimes (1996: p. 25) maintained the specimens “Surinam: im Reservat der Zanderij I, die nummerierten Baume n. 102 (Herb. [Acad. Rhenotraiect. J n. 1529, im Dec. 1915, und n. 4350 bl. im Juli 1919) und n. 141 (Herb. n. 4357, bl. im Juli 1919.)” as syntypes. In the present work, the specimen *Forest Bureau 4357* (IAN49436) is designated as lectotype (Art. 9.3, Turland et al. 2018).

**Figure 5.**
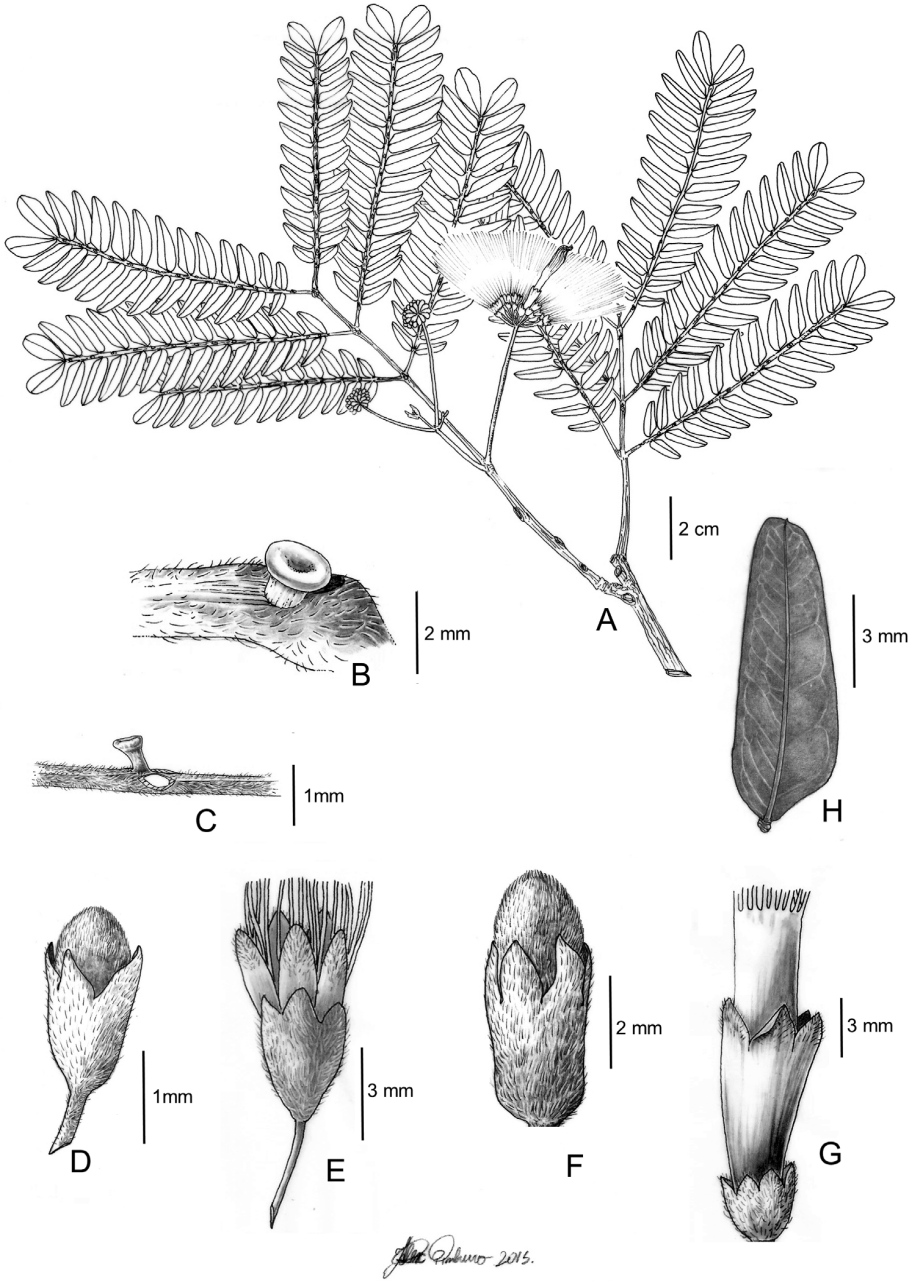
*Hydrochoreagonggrijpii* (Kleinhoonte) Barneby & J.W. Grimes **A** branch with inflorescences **B, C** extra-floral nectaries **D** peripheral flower bud **E** peripheral flower **F** terminal flower bud **G** terminal flower **H** leaflet. **A–H** from *Fróes 28045*. Illustration by Alex Pinheiro.

##### Selected specimens examined.

Brazil, Amazonas: Presidente Figueiredo, Cachoeira do boto, 21 September 2007, *Carvalho-Sobrinho et al 1632* (RB). Colombia, Vaupés: Mitú and vicinity, lower Río Kubiyú, along river, 26 September 1976, *J.L. Zarucchi s.n.* (K). Suriname. Plantas de Tafelberg (Table Mountain), 10 August 1944, *Maguire 24273* (RB). Venezuela, Bolivar: Distrito Piar, gallery forest bordering savana, vicinity of Guadequen (Buadequen), Río Acanán (affluent of Río Carrao), Cerros Los Hermanos, 20 May 1986, Lat 5°26'N, Long 62°17'W, alt 470 meters, *J.A. Steyermark et al. 131865* (NY).

#### 4. Hydrochorea leucocalyx

Taxon classificationPlantaeFabalesFabaceae

(Britton & Rose) Iganci, M.V.B. Soares & M.P. Morim comb. nov.

77D73681-6358-51F0-A90A-DEB01B38B288

urn:lsid:ipni.org:names:77303828-1

Fig. 2A

Balizia leucocalyx (Britton & Rose) Barneby & J.W. Grimes, in Mem. New York Bot. Gard. 74(1): 85. 1996.

##### Basionym.

*Samanealeucocalyx* Britton & Rose, N. Amer. Fl. 23: 34. 1928.

##### Type material.

Mexico. Tabasco, El Limon, *J. N. Rovirosa 976* (lectotype, designated by Barneby and Grimes 1996, p. 36, as holotype, here corrected: US [US13198371] digital image!, clastotypus (fragm. + photo): NY [NY00003824] digital image!).

##### Distribution and habitat.

Belize, Guatemala, Honduras, Mexico. *Hydrochorealeucocalyx* occurs in wet tropical forests, often along riverbanks, seldom in anthropogenic pastures, up to 400 m elevation (Barneby and Grimes 1996).

##### Notes.

Amongst the species of *Hydrochorea*, *H.leucocalyx* is one of the few that does not occur in Amazonia. It has affinities with the new species described in this treatment (see *H.uaupensis*) and is mainly distinguished by the lomentiform indehiscent fruit (vs. the cryptoloment in *H.uaupensis*). Barneby and Grimes (1996) recognised the specimen *J. N. Rovirosa 976* as holotype, but in the species protologue (Britton and Rose 1928), the authors did not indicate the herbarium where the type specimen was deposited. Thus, following Art. 9.10 of the International Code of Botanical Nomenclature (Turland et al. 2018), the specimen *J. N. Rovirosa 976* (US13198371) is here corrected to lectotype.

##### Selected specimens examined.

Honduras: 7 September 1932, *W.S. Schipp 1024* (K). Mexico, Chiapas: km 12 carretera Pénjamo-Chancalá, 8 June 1960, *J.P. Chavelas et al. s.n.* (K).

#### 5. Hydrochorea marginata

Taxon classificationPlantaeFabalesFabaceae

(Spruce ex Benth.) Barneby & J.W. Grimes, Mem. New York Bot. Gard. 74(1): 29. 1996.

8172C4D2-1911-5DD1-9299-9AD42912A6C2

Figs 2A, D , 6

##### Basionym.

*Pithecellobium* [as *Pithecolobium*] *marginatum* Spruce ex Benth., Trans. Linn. Soc. London 30: 586. 1875.

##### Type material.

Brazil, Barra, by a stream [Prov. Rio Negro], *Spruce 1658* (lectotype, designated by Barneby and Grimes 1996, p. 31: K [K000528011]!; isolectotypes: E [E00313848] digital image!, F [V0058733F] digital image!], G [G275450] digital image!, P [P03094432] digital image!, P [P03094430] digital image!).

**Figure 6.**
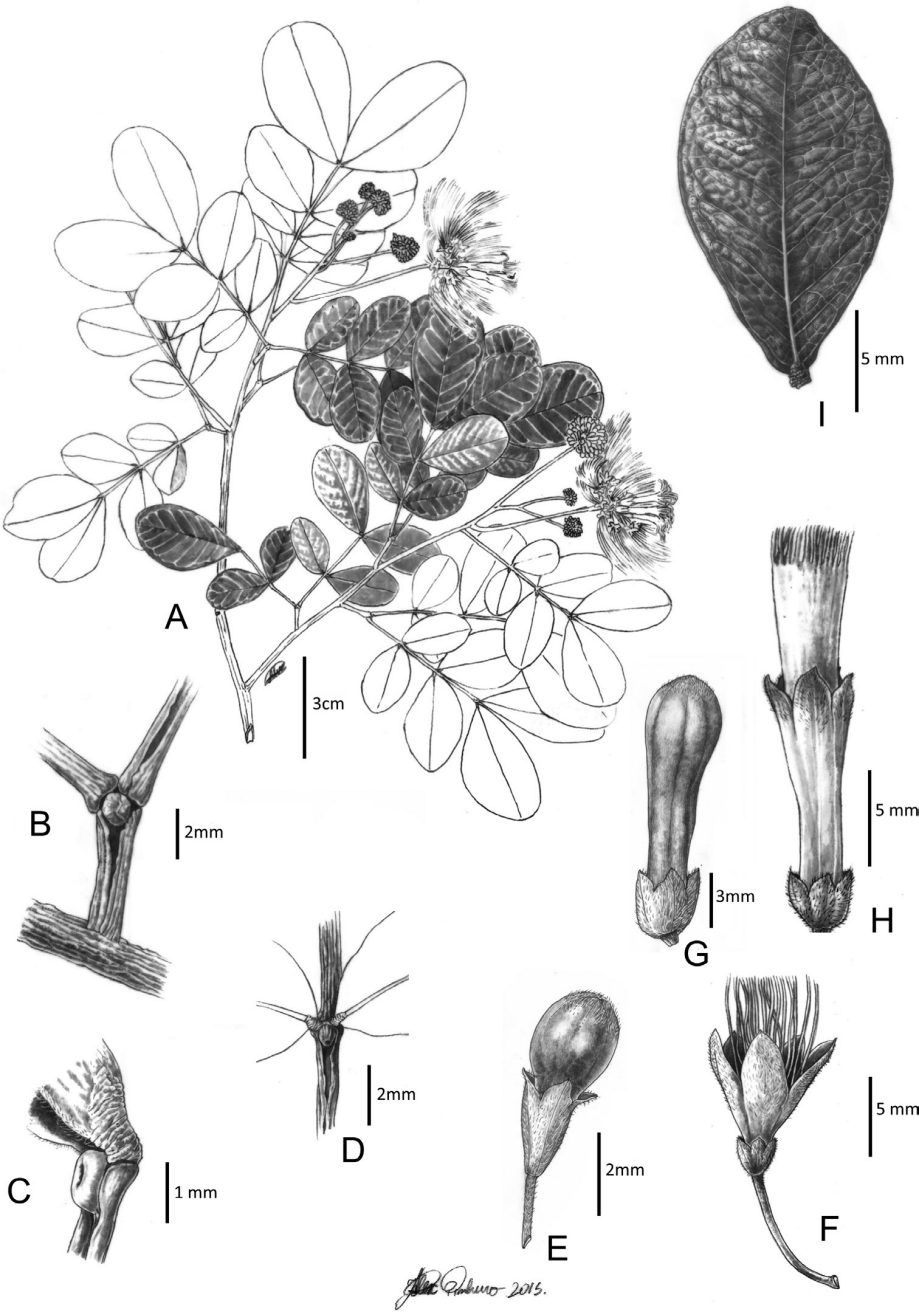
*Hydrochoreamarginata* (Spruce ex Benth.) Barneby & J.W. Grimes **A** branch with inflorescences **B–D** extra-floral nectaries **E** peripheral flower bud **F** peripheral flower **G** terminal flower bud **H** terminal flower **I** leaflet. **A–D, F, H, I** from *A. Carlos et al. 066***E, G** from *C. Ferreira et al. 7260*. Illustration by Alex Pinheiro.

##### Distribution and habitat.

Brazil and Venezuela. *Hydrochoreamarginata* occurs in Amazonia, in flooded areas and along riverbanks and lake shores.

##### Notes.

Barneby and Grimes (1996) considered *H.marginata* to comprise three varieties, H.marginatavar.panurensis (Benth.) Barneby & J.W. Grimes, H.marginatavar.scheryi Barneby & J.W. Grimes, and Hydrochoreamarginatavar.marginata. Hydrochoreamarginatavar.panurensis is recognized at the species level in this treatment, and H.marginatavar.scheryi is placed as a synonym of *H.panurensis* because we identified no morphological diagnostic characters that support them as independent taxa. Both these taxonomic decisions are discussed under *H.panurenis*.

##### Selected specimens examined.

Brazil: Amazonas, Rio Negro between Moreira and Rio Arirahá, 13 October 1971, *G.T. Prance 15206* (NY).

#### 6. Hydrochorea obliquifoliolata

Taxon classificationPlantaeFabalesFabaceae

(De Wild.) E.J.M. Koenen comb.nov.

B04306BD-20E4-5F5B-8322-447CFD1C201F

urn:lsid:ipni.org:names:77303829-1

Figs 2A , 3L

Pithecellobium obliquifoliolatum (De Wild.) J. Léonard, in Compt. Rend. Sem. Agric. Yangambi Comm. No. 67, 868 (1947).
Pithecellobium obliquifoliolatum (De Wild.) Aubrév., Fl. Forest. Soudano-Guin. 290 (1950), in obs., Aubrev. in Not. Syst., ed. Humbert, xiv. 57 (1950) nom. illeg.;
Arthrosamanea obliquifoliolata (De Wild.) G.C.C. Gilbert & Boutique, Fl. Congo Belge & Ruanda-Urundi iii. 194 (1952).
Cathormion obliquifoliolatum (De Wild.) G.C.C. Gilbert & Boutique, Bull. Soc. Roy. Bot. Belgique 90: 309 (1958).

##### Basionym.

*Albiziaobliquifoliolata* De Wild., Bull. Jard. Bot. État Bruxelles 7: 253 (1920).

##### Type material.

Democratic Republic of the Congo, Congo Belge, Eala, *Laurent 1823* (lectotype, designated here: BR [BR0000008916334]!; isolectotype: BR [BR0000008916662]!).

##### Description.

**Trees** up to 30 m in height and up to 1m DBH, the bark with both small scattered and long transverse linear lenticels, the indumentum consisting of a dense rusty to golden-brown pubescence covering the young twigs, petiole and primary rachis, with more sparse pubescence on peduncles and pinna-rachises except for dense rows of hairs at the margins of the otherwise glabrous canaliculate adaxial side of the pinna rachises, often also the canaliculate primary rachis of the leaf sparsely pubescent to glabrous adaxially. **Stipules** linear deltoid to falcate, 2–3 mm long, adaxially glabrous except at apex, densely pubescent, caducous. **Leaves** with (1–)2(–3) pairs of pinnae, petiole pulvinate and slightly flattened at base, (1.5–)2–3.5(–4.5) cm long, with a sessile concave circular to triangular nectary at the apex, c. 0.8–1.5 mm in diameter, rachis usually canaliculate adaxially, (0–)1.5–3(–6) cm long, if the leaf 3-jugate then usually with an inter-pinnal nectary, similar to the petiolar one, in between the middle pair of pinnae, apical nectary usually lacking, pinnae distinctly pulvinate, and usually with minute paraphyllidia at the apex of the pulvini, pinna-rachises canaliculate adaxially, the groove glabrous, c. (3–)4–11(–15) cm long, with short stipitate circular to elliptical cupular or trumpet-shaped nectaries c. (0.2–)0.5–1 mm in diameter. Leaflets in (3–)5–7(–8) pairs per pinna, subsessile on a c. 0.5 mm long pulvinule, widely spaced so that the margins do not overlap, bicolorous, dark green and shiny above, pale dull green beneath, rhomboid and often distinctly curved towards pinna apices, base asymmetrically obtuse or slightly oblique and the apex rounded or shallowly emarginate, sometimes mucronate, (1.4–)2.2–3.5(–4.7) × (0.6–)1.1–1.8(–2.2) cm, except the apical pair that is asymmetrically elliptic with oblique base and emarginate apex, (1.8–)2.7–4.8(–5.5) × (1.0–)1.5–2.5 cm, venation pinnate with (6–)12–16(–18) secondary veins brochidodromous, prominent on both surfaces or prominulous to slightly sunken on upper surface, and reticulate tertiary venation, often prominent on upper surface, obscure beneath, margins and midrib ciliate on both surfaces, lamina glabrous but for a few short scattered appressed hairs. **Inflorescences** (10–)15–20 flowered umbelliform capitula, on long slender peduncles arising 1–2 from axillary buds of coeval or caducous leaves, held above the foliage, the axillary meristems usually not continuous beyond the peduncles and aborted prior to fruit set, dimorphic with a single enlarged terminal flower and often one dispositioned peripheral flower c. 0.5 cm below the others, on peduncles 4–8(–12) cm long. Bracts linear to spatulate, sometimes bilobed at apex, c. 2–3.5 × 0.5 mm, pubescent with longer hairs at apex. Peripheral flowers on pedicels 2–6 mm long, calyx pentamerous, green to greenish white, c. 3–4 mm long, the deltoid lobes c. 0.5 × 0.5 mm, glabrous, corolla pentamerous, green to greenish white, c. 5–6 mm long, the lobes c. 2–3 × 2 mm, glabrous or with short white hairs around the apices of the lobes, androecium consisting of c. 20–30 stamens, c. 2.1–2.5 cm long, the filaments white to pale green at apex, fused into a tube c. 3 mm long, with dorsifixed pale yellowish green anthers, pollen in 16-celled plano-compressed disc-shaped polyads, pistil c. 2.5–2.8 cm long, ovary c. 3 mm long, pubescent in upper half, the pale green to white style emerging from it at an angle of c. 45°, with a green funnel-shaped stigma, extending beyond the stamens. Terminal flower sessile to subsessile, similar to peripheral flowers but broadly campanulate and larger, calyx c. 3.5–5 mm and corolla c. 7.5–9 mm long, the filaments thicker and staminal tube c. 8–10 mm long, exserted well beyond the corolla tube. **Pods** falcate and weakly articulated, base often tapering into a c. 5 mm long stipe, (3–)6–12 seeded with a thin papery fruit wall and slightly thickened rim, dark brown to black outside when ripe, light brown inside, (3.7–)5.5–9.6 × 1.2–1.4 cm, breaking up into 1-seeded articles 0.4–0.7(–1.0) cm long, the basal and apical articles up to 1 cm long, seed c. 6.5 × 4.5 × 0.5 mm, the testa hard, light brown with a darker brown closed elliptic pleurogram, c. 4 × 2 mm.

##### Distribution and habitat.

Gabon, Central African Republic, Congo-Brazzaville, Democratic Republic of the Congo. *Hydrochoreaobliquifoliolata* occurs in the Congo Basin, and is a species of swamp forests, seasonally inundated forests and riverbanks.

##### Notes.

The similarities to *Cathormionrhombifolium*, the other African species that is here transferred to *Hydrochorea*, are discussed below.

##### Selected specimens examined.

Gabon: Ogooué-Lolo, road Okondja to Bambidie and Lastoursville, 21 km SW of Okondja, 7 February 2008, *J.J. Wieringa 6519* (BR, K). Democratic Republic of Congo: Yafunda, rive guache, près d’Isangi, 8 September 1938, *J. Louis 11175* (BR). Boendu, August 1938, *Du Bois 904* (BR), *G. Couteaux 55* (BR). Bolomba, 7 November 1957, *C. Évrard 2746* (BR). Bongoy, 4 January 1958, *C. Évrard 3191* (BR). Botsima, route station-village, 28 January 1991, *J.B.M.M. Dhetchuvi 321* (BR). Yangambi, île Tutuku en face du plateau de l’Isalowe, 3 January 1940, *R.G.A. Germain 87* (BR). Bokondji, 28 September 1959, *De Wanckel 162* (BR).

#### 7. Hydrochorea panurensis

Taxon classificationPlantaeFabalesFabaceae

(Spruce ex Benth.) M.V.B. Soares, M.P. Morim & Iganci comb. nov.

10C8808B-B354-5266-BEA8-4C4BECB054E5

urn:lsid:ipni.org:names:77303830-1

Figs 2A , 7

Hydrochorea marginata var. panurensis (Benth.) Barneby & J.W. Grimes, Mem. New York Bot. Gard. 74: 32. 1996. Type: based on Pithecellobiumpanurense Spruce ex Benth., syn. nov.
Hydrochorea marginata var. scheryi Barneby & J.W. Grimes, Mem. New York Bot. Gard. 74: 32. 1996. Type: Venezuela, at Sanariapo, Territorio Federal Amazonas, *Llewellyn Williams 15953* (lectotype first step, designated by Barneby and Grimes 1996, p. 32, as holotype, here corrected: F; lectotype second step, here designated: F [V0058706F], digital image!; isotypes: F [V0058707F] digital image!), G [G00365761] digital image!, GH [GH00060404] digital image! K [K0005279] digital image!, MO [MO954357] digital image!, MO [MO954358] digital image!), NY [NY00334620] digital image! US [US00000401] digital image!, US [US00385625] digital image!, VEN [VEN2237] digital image!, syn. nov.

##### Basionym.

*Pithecellobium* [as *Pithecolobium*] *panurense* Spruce ex Benth., Trans. Linn. Soc. London 30: 586. 1875.

**Figure 7.**
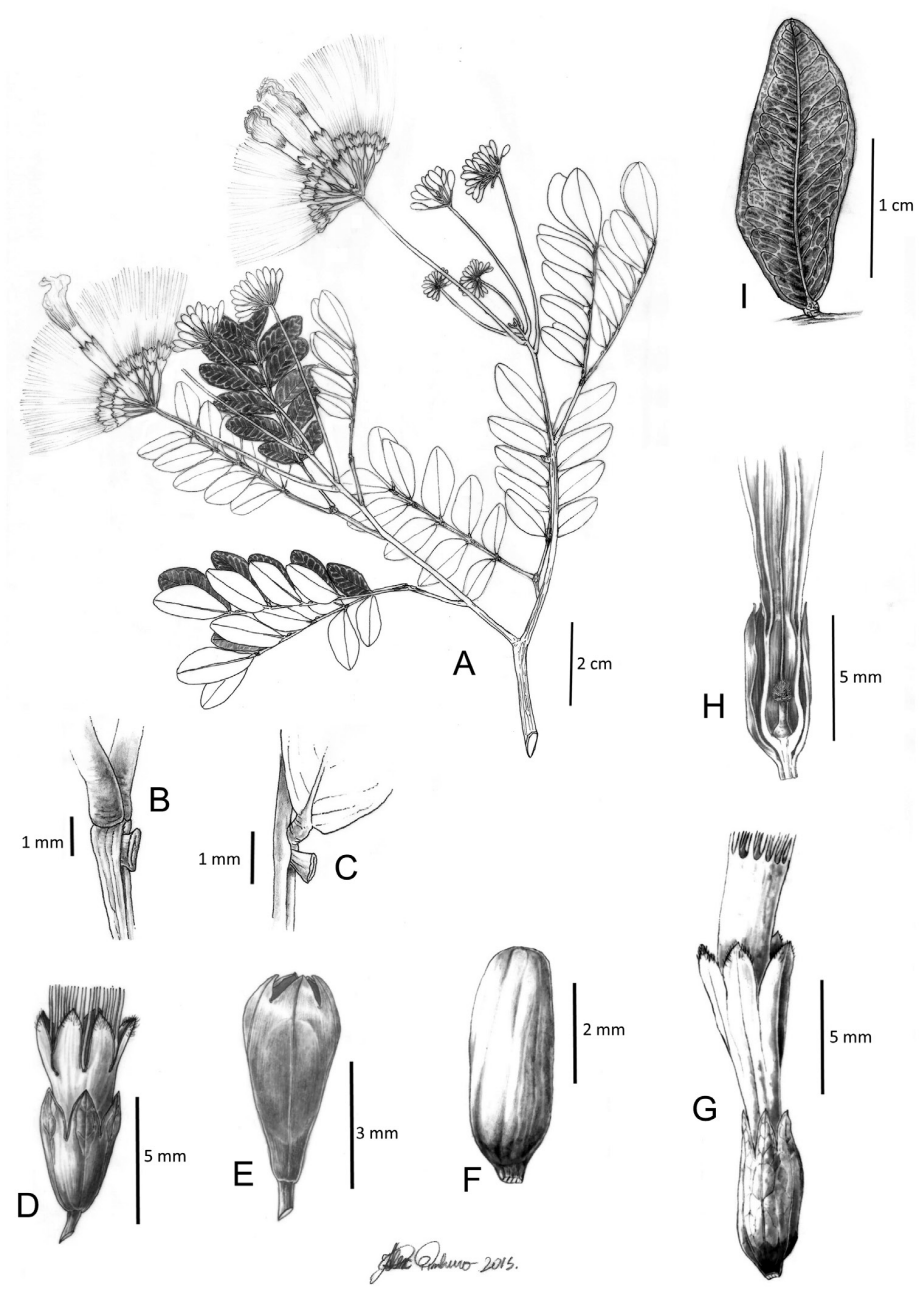
*Hydrochoreapanurensis* (Spruce ex Benth.) M.V.B. Soares, M.P. Morim & Iganci **A** branch with inflorescences **B, C** extra-floral nectaries **D** peripheral flower **E** peripheral flower bud **F** terminal flower bud **G** terminal flower **H** terminal flower longitudinal section **I** leaflet. **A–I** from *Wurdack & Adderley 43618*. Illustration by Alex Pinheiro.

##### Type material.

Brazil, in silvis ‘Gapó’ ad flumen Uaupés prope Panuré, prov. do Alto Amazonas, *Spruce 2425* (lectotype first step, designated by Barneby and Grimes 1996, p. 32, as holotype, here corrected: K; lectotype second step, here designated: K [K000528008]!; isolectotypes: E [E00313845] digital image!, F, [V0058739F] digital image!, G [G00365356] digital image!, G [G00365687] digital image!, GH [GH00063965] digital image!, K [K000528007]!, NY [NY334690], P [P03094382] digital image!, P [P03094383] digital image!, RB [RB00708599]!).

##### Distribution and habitat.

Brazil, Venezuela. *Hydrochoreapanurensis* occurs in seasonally flooded Amazonian sites along rocky stream banks and ecotone with gallery forests.

##### Notes.

Ducke (1949) considered *Pithecellobiumpanurense* as a form of *P.marginatum*, considering the vegetative characters to be more important taxonomically than the differences between the flowers of those taxa. Barneby and Grimes (1996) moved *P.panurense* to *Hydrochorea* as H.marginatavar.panurensis. However, *Hydrochoreapanurense* has diagnostic floral characters which distinguish it from *H.marginata*. The floral buds of *H.panurense* have the corolla covered by the calyx; the flowers are glabrous and the terminal flower has a tubular calyx (vs. calyx not covering the corolla in the floral buds, pubescent flowers, and calyx of the terminal flower campanulate in *H.marginata*). Barneby and Grimes (1996) also described H.marginatavar.scheryi, distinguishing it from H.marginatavar.panurensis only by the pedicel of the peripheral flowers, this 4–5 mm long. (vs. 6.5–13 mm long in H.marginatavar.panurensis). Barneby and Grimes (1996) emphasized, however, that this is the only character to distinguish between the two taxa. In the present study we consider H.marginatavar.scheryi to be a synonym of *H.panurensis*.

A second step lectotypification is designated here for both *H.panurensis* and H.marginatavar.scheryi, since the material that Barneby and Grimes (1996) recognised as holotypes consists of two sheets in both cases and they did not select lectotypes from amongst these sheets (Art. 9.10,9.17; Turland et al. 2018).

##### Selected specimens examined.

Brazil, Amazonas: Barcelos, Serra do Araçá, Rio Araçá à 13 h de Barcelos, 28 July 1985, *Silva 389* (INPA); São Gabriel da Cachoeira. Margem do Rio Içana em direção a comunidade Camarão, 0°48'35.8"N, 67°32'10"W, 19 July 2012, *Morim*, *M.P.*, *Iganci*, *J.R.V*, *Bonadeu F & Koenen*, *E. 563* (RB, Z). Venezuela, Amazonas: Rio Casiquiare, 11 November 1959, *Wurdack & Adderley 43407* (IAN).

#### 8. Hydrochorea pedicellaris

Taxon classificationPlantaeFabalesFabaceae

(DC.) M.V.B. Soares, Iganci & M.P. Morim comb. nov.

71652CE9-BE1B-57EE-B55B-E1AE26D7B1B1

urn:lsid:ipni.org:names:77303831-1

Figs 2A, B , 3F-I

Balizia pedicellaris (DC.) Barneby & J.W. Grimes, in Mem. New York Bot. Gard. 74(1): 85. 1996.
Albizia pedicellaris (DC.) L. Rico, in Novon 9(4): 555. 1999.

##### Basionym.

*Ingapedicellaris* DC., Prodr. 2: 441. 1825.

##### Type material.

French Guiana, “...in Cayenna” (lectotype, designated by Barneby and Grimes 1996, p. 36, as holotype, here corrected: G-DC = F Neg. 6972, digital image!).

##### Distribution and habitat.

Bolivia, Brazil, Colombia, Ecuador, French Guiana, Guyana, Peru, Suriname, Venezuela. *Hydrochoreapedicellaris* occurs in non-inundated primary rainforest in Amazonia, in the lowlands of the Atlantic Rainforest, and in gallery forests, up to 200 m elevation, and occasionally at 700–800 m elevation in Bolivia, Ecuador and eastern Brazil (Barneby and Grimes 1996).

##### Notes.

*Hydrochoreapedicellaris* is the only species of the genus that occurs in a range of environments including areas of the Brazilian Atlantic Forest. It has an affinity with *H.elegans* (see comment under that species), but when it is in fruit it is easily recognized by its follicular dehiscence, and an exocarp with deep, transversal fissures. Barneby and Grimes (1996) recognised a specimen in the G-DC herbarium as holotype, but in the species protologue (De Candolle 1825), the author did not indicate a type specimen. Thus, the specimen at G-DC should be considered a lectotype, here corrected (Art. 9.10; Turland et al. 2018).

##### Selected specimens examined.

Bolivia, La Paz: Province of Larecaja, Tuiri, 12 September 1989, *B. Krukoff 10886* (K). Brazil, Amazonas: São Gabriel da Cachoeira, Rio Içana, na comunidade Camarão, terra firme, 0°37'23"N, 67°26'57"W, 20 July 2012, *Iganci*, *J.R.V*, *Morim*, *M.P.*, *Bonadeu F.*, *Koenen*, *E.* 870 (RB); Espírito Santo: Linhares Fragmento em frente a casa do Reis, Sítio Santo Domingo, Restinga arbórea de cordões arenosos, 19°21'6"S, 39°43'31"W, 13 March 2007, *R.D. Ribeiro et al. 812* (RB). Guyana: Territorio Federal Delta Amacuro, 29 May 1964, *L.M. Berti 225* (K). Peru: Palcazú, Pasco Oxapampa, localidad Mayro, 20 May 2010, *R. Vásquez et al. 36546* (K). Suriname: Zenderij, November 1944, *M. Koeleroe 237* (RB). Venezuela: Altiplanicie de Nuria, 15 July 1960, *J.A. Steyermark 86335* (K).

#### 9. Hydrochorea rhombifolia

Taxon classificationPlantaeFabalesFabaceae

(Benth.) E.J.M. Koenen comb. nov.

492FC025-6480-5E32-AF87-6D60DD90AD63

urn:lsid:ipni.org:names:77303832-1

Figs 1A , 3M

Feuilleea rhombifolia (Benth.) Kuntze, Revis. Gen. Pl. 1: 189 (1891).
Cathormion rhombifolium (Benth.) Keay, Kew Bull. 8(4): 489 (1953).

##### Basionym.

*Albiziarhombifolia* Benth., London J. Bot. 3: 87 (1844).

##### Type material.

Guinée, Conakry, *Heudelot 735* (lectotype designated here from amongst the syntypes: K [K000043955]!; isolectotypes: K [K000043954]!, K [K000043949]!, P [P00418271] digital image!, P [P00418272] digital image!, P [P00418270] digital image!).

##### Description.

**Trees** or **shrubs** up to 12 m tall, the young stems, all leaf-axes and peduncles puberulent-tomentulose with rusty brown hairs. **Stipules** deltoid, c. 1 mm long, puberulent-tomentulose, caducous. **Leaves** with 2–3 pairs of pinnae, petiole pulvinate, ventrally flattened above pulvinule and with central groove in upper half, 2–3.5(–8.5) cm long, rachis ventrally grooved, 1.5–4(–12.5) cm long, pinna rachises pulvinate, ventrally grooved, (3.2–)4–6(–12) cm long. Nectaries present at the petiole apex just below the first pair of pinnae as well as just below each further pair of pinnae, sessile or shortly stipitate on stipe to 0.5 mm, cupular or sometimes concave, circular and 0.8–2.2 mm in diameter, and between the upper 2–3 pairs of leaflets, trumpet-shaped and then on a short stipe 0.5 mm or cupular and (sub)sessile, the lower ones circular and the upper ones elliptical, 0.8–1.5 × 0.8–1.1 mm. Minute paraphyllidia sometimes present at the apex of the pinna-pulvinus. Leaflets in 4–6 pairs per pinna, closely spaced, bicoloured leaflets often with partly overlapping margins, bright green above and pale green beneath, dull on both surfaces, rhomboid with a pulvinate sessile oblique base and rounded to slightly emarginate apex, increasing in size towards pinna apex, (1.1–)1.7–3.5(–5.1) × (0.5–)1.2–1.8(–2.3) cm, except for the apical pair which has a less oblique to nearly acute base, (2.1–)2.5–4.5(–5.7) × (1.1–)1.5–2.5(–3.2) cm; venation pinnate with 8–12(–18) secondary veins brochidodromous, tertiary venation reticulate, prominulous on both surfaces, midribs ciliate on both sides, the lower leaflet surface pilose with a variable density of brownish to white hairs, rarely almost glabrous, sometimes villose particularly near the midrib giving a rusty orange-brown appearance. **Inflorescences** umbelliform capitula, axillary to co-eval leaves on peduncles (4.5–)5–9.5 cm long, dimorphic with 6–16 peripheral flowers and 1–2 terminal flower(s) with elongated exserted staminal tubes. Bracts spatulate, c. 1.8 mm long, puberulent with minute rusty hairs, caducous. Peripheral flowers on pedicels of 1–4 mm, calyx pentamerous, white, 3–3.5 mm long, fused, the deltoid lobes 1–1.3 mm long, glabrous or with few minute hairs, corolla pentamerous, white, 6–8 mm long, fused in the lower half, glabrous, pilose to villose in the upper half, androecium 1.6–2.3 cm long, consisting of 20–28 stamens with white filaments fused at the base into a short tube of c. 2 mm, anthers dorsifixed, pollen in 16-celled plano-compressed disc-shaped polyads, gynoecium with a c. 2 mm long ovary, pubescent on the upper half, the 1.6–2.5 cm long white style emerging from it at an angle of c. 45°, with a funnel-shaped stigma, extending beyond the stamens. Terminal flower(s) similar but larger and more robust in appearance, calyx c. 4.5 mm long with c. 1.5 mm long lobes, corolla c. 9 mm long, androecium with 30–36 stamens that are thicker and fused into a tube 7–10 mm long, exserted well beyond the corolla tube, and with a sunken nectariferous disk below the base of the ovary, gynoecium otherwise similar to that of the peripheral flowers. **Pods** straight to falcate, 6–12-seeded with a thin papery fruit wall and thickened rim, dark brown outside when ripe, whitish grey inside, (4.5–)7–12.5 × 1.4–1.9 cm, breaking up into 1-seeded articles 0.6–1.1 cm long, seed c. 7 × 4.5 × 2 mm, the testa hard, light brown with a wide lighter brown closed pleurogram.

##### Distribution and habitat.

Known from the tidal riverine systems near the coast from Senegal to Sierra Leone. *Hydrochorearhombifolia* occurs often abundantly, in permanent or tidal swamp forest, including on the edge of mangrove swamps, and in gallery forests.

##### Notes.

Bentham (1844) described *Albiziarhombifolia*, before designation of holotypes was required by the International Code of Botanical Nomenclature. Keay (1953) made the new combination *Cathormionrhombifolium* and cited the holotype as being at Kew. However, there are three specimens of *Heudelot 735* at K, the type that was cited by Bentham, leaving it ambiguous as to which one of these represents the holotype. Therefore, the specimen from Herbarium Benthamianum (the oldest deposited specimen dating to 1854) is here designated as a lectotype: it has leaves and flowers, and is more richly annotated than the other two specimens.

*Hydrochoreaobliquifoliolata* and *H.rhombifolia* are morphologically very similar and have sometimes been confused in herbaria, despite their clearly different geographical distributions. The species are readily separated by the darker appearance of the leaflets of *H.obliquifoliolata*, which have a distinct shine on the upper surface and the lower surface usually (sub-)glabrous (vs. a usually rusty pilose lower leaflet surface in *H.rhombifolia*). The leaflets of *H.rhombifolia* are also more closely spaced than those of *H.obliquifoliolata*, the latter not having overlapping margins. Furthermore, the flower colour of the two species is clearly different (as per the key), a characteristic which remains apparent when comparing dried flowering specimens in the herbarium, and the corolla lobes of *H.obliquifoliolata* are glabrous or with a few short apical hairs (vs. pilose to villous on the upper half in *H.rhombifolia*).

##### Selected specimens examined.

Sierra Leone: Mange, 7 February 1939, *F.C. Deighton 3618* (K), Rokupr, 25 May 1953, *F.C. Deighton 5925* (K), Kasanko (Mafore), 3 December 1950, *T.S. Jones 52* (K), near Tassin and Kukum, 17 January 1892, *G.F. Scott Elliot 4418* (K); Guinée-Bissau: Gabu, Ponte do rio Colufe, 10 June 1949, *Espirito Santo 2500* (K).

#### 10. Hydrochorea uaupensis

Taxon classificationPlantaeFabalesFabaceae

M.P. Morim, Iganci & E.J.M. Koenen sp. nov.

3BE35DAE-0FD1-5E19-B087-AEDA50DF6775

urn:lsid:ipni.org:names:77303833-1

**Figure 8.**
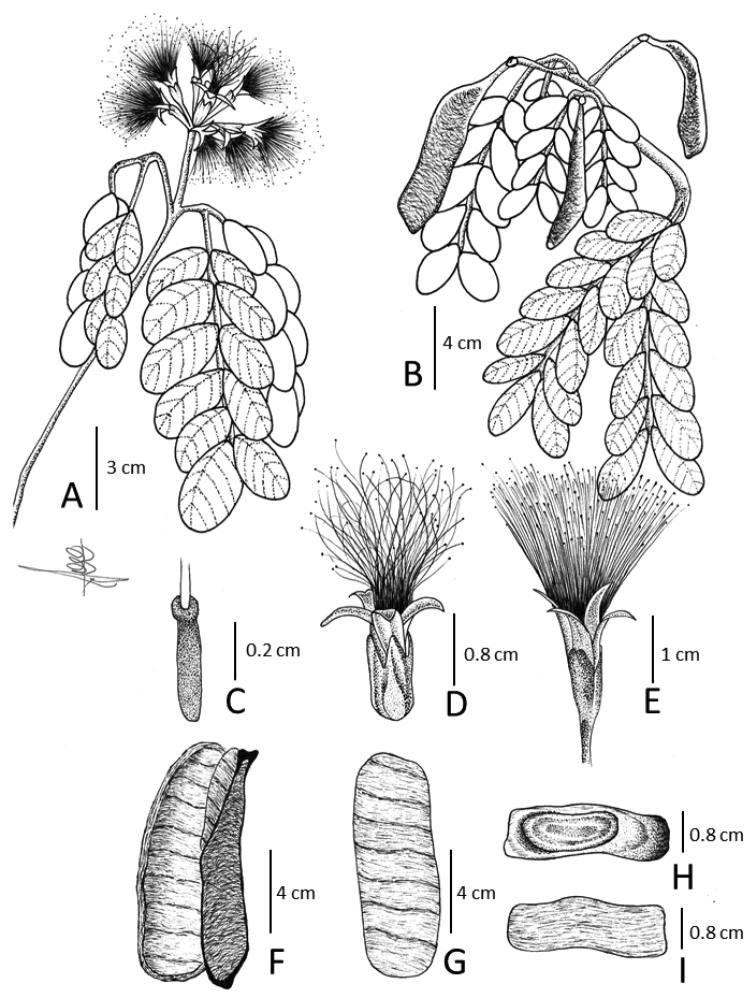
*Hydrochoreauaupensis* M.P. Morim, Iganci & E.J.M. Koenen **A** branch with inflorescences **B** branch with fruits **C** ovary **D** terminal flower **E** peripheral flower **F** dehisced fruit **G** detail of fruit endocarp forming 1-seeded articles **H, I** monospermous articles. **A–I** from *M.P. Morim et al. 577*. Illustration by João Augusto Castor Silva.

Figs 2A, E-G , 3J, K , 8

##### Diagnosis.

*Hydrochoreauaupensis* is morphologically similar in appearance to *H.leucocalyx* (Britton & Rose) Iganci, M.V.B. Soares & M.P. Morim by its leaflets and inflorescence, however it differs by having a red or green calyx, pink corolla, 1–2 pairs of pinnae, and crypto-lomentiform fruits (vs. white calyx and corolla, 3–5(–6) pairs of pinnae and indehiscent fruits in *H.leucocalyx*).

##### Type material.

Brazil, Amazonas, São Gabriel da Cachoeira. Igarapé Tibuari, afluente do Vaupés 0°05'5"N, 67°23’16"W, 23 July 2012, fl. and fr., *M.P. Morim*, *J.R.V. Iganci*, *F. Bonadeu*, *E. Koenen 577* (holotype: RB [RB00728413]!; isotypes: HUEFS!, INPA!, K!, MBM!, MG!, MO!, NY!, PEL!, S!, US!, Z!).

##### Description.

**Trees** 2–6 m tall, trunk not observed, partially underwater during seasonal inundation. Branches, leaf axes and peduncles sparsely pubescent to glabrescent. **Stipules** linear, to 1.2 cm long, densely pubescent on outer surface. **Leaves** with 1–2 pairs of pinnae; petiole including pulvinus 1.5–4.5 cm, cylindrical; rachis 0 or 3–4(–9) cm, glabrous, canaliculate; extrafloral nectaries borne between first or both pairs of pinnae, sessile, patelliform and smaller nectaries usually present between the leaflet pairs; pinnae 3–5 jugate; leaflets subsessile on pulvinules, chartaceous, c. 2–4(–6) × 1.5–2(–3) cm, rhomboid or ovate, apex emarginate or obtuse, sometimes with a minute mucro, base asymmetrically oblique to acute; adaxial and abaxial surfaces glabrous, discolorous, adaxial surface sometimes lustrous; venation pinnate with c. 11–17 secondary veins, tertiary venation reticulate and prominent on both surfaces when leaflets dry. **Inflorescences** dimorphic, umbelliform with 7–10 peripheral flowers and an enlarged sessile terminal flower, peduncle 4–6(–8) cm. Bracts and bracteoles not seen. Flowers with a reddish or green calyx, pink corolla and white filaments, the flower buds oblong, ca. 8 mm long, with the corolla concealed by the calyx prior to anthesis, peripheral flowers on pedicels 0.7–1.5 cm, calyx campanulate, c. 9 mm long, 5-angulate due to prominently raised veins, sparsely puberulent or ciliate at the apex of the lobes, corolla tubular, with a prominulous midvein on the lobes, c. 1.5 cm long, sparsely puberulent on the upper half of the lobes, stamens c. 50–60, the filaments white, c. 3 cm long, exserted from the corolla ca. 2 cm; ovary glabrous, 3–4 mm, sub-truncate to truncate at the apex, style 3.5–4 cm, stigma funnel-shaped; terminal flower similar to peripheral flowers but more robust and c. 5 mm wide at base, calyx c. 1.2 cm long, corolla 1.6 cm long, stamens ca. 75, ca. 3.5 cm long. **Pods** typically 1–3 per infructescence, crypto-lomentiform, up to 15-seeded, oblong, slightly curved, lignescent, c. 9.5 × 2.5 cm excluding a ca. 5 mm long mucro, dehiscence follicular, the smooth exocarp and transversely fibrous mesocarp continuous, the endocarp septate, enveloping the seeds which are released in monospermous articles. **Seeds** not seen in mature state, oblong, c. 1.6 × 0.4–0.7 cm, pleurogram extending from apex to base, c. 1.3 × 0.3–0.4 cm, closed.

##### Distribution and habitat.

Brazil. Known only from the Upper Rio Negro region in the Brazilian Amazon (Amazonas state), in seasonally inundated “campinarana” vegetation.

##### Phenology.

Flowering and fruiting in July.

##### Etymology.

The specific epithet refers to the type locality, near the River Uaupés, in the state of Amazonas, Brazil. The indigenous people living in this area (e.g., the Tucanos) were known as Uaupés, and later the river took the same name.

##### Notes.

*Hydrochoreauaupensis* is only known from Amazonas state, Brazil, where it was collected at “Igarapé Tibuari”, in the municipality of São Gabriel da Cachoeira, during fieldwork in July 2012. The species grows in open vegetation on white sand, known in Brazil as campinarana in the Amazon Domain. During times of flood, only the treetops are exposed above the water line. A second herbarium collection from close to the type locality (Rio Tourí, afl. do Rio Negro, igapó; *R.L. Fróes 28691*, IAN [IAN78279]), of which we have only seen an image, is here tentatively included under *H.uaupensis* because the fruit and leaves match the type material and the flowers are described as pink on the specimen label. Since these two occurrence records are close to the borders with Colombia and Venezuela, the species is to be expected in those two countries.

The phylogenetic position of *H.uaupensis*, as the sister lineage of the clade composed of *Hydrochorea* sensu Barneby and Grimes (1996) and the African *Hydrochorea* spp., provides ample support for this as a distinct taxon and a species new to science, as it does not form a sister pair with any other known species. Furthermore, this phylogenetic position is in line with the fruit morphology of the species being intermediate between *Balizia* and *Hydrochorea*, adding further support, along with the paraphyly of *Balizia*, for not maintaining these as distinct genera.

##### Conservation status.

Data deficient. The species is known only from two adjacent localities in the Upper Rio Negro region of Amazonas state, Brazil. More collections are needed to assess the species’ conservation status.

## Conclusion

Our results provide significant advances in the generic delimitation of *Hydrochorea* and related taxa, as well as broadening our understanding of ongoing diversification in these taxa. The uncertain phylogenomic position of *Jupunbamacradenia*, and other species of *Jupunba*, sharing a relatively large number of incompletely sorted genes with *Hydrochorea*, leads to further difficulties in our ability to delimit genera in a group where classification has been notoriously unstable. Nevertheless, given the complex evolutionary patterns across the genome presented by the Jupunba clade taxa, we decided to use morphology as our main guide for taxonomic decisions, re-circumscribing *Hydrochorea* to include ten species to account for the paraphyly of *Balizia*, while incomplete lineage sorting surrounding the divergence between *Hydrochorea* and *Jupunba* does not falsify these two genera as natural groups. Furthermore, not transferring all these species to *Jupunba*, although a cautious decision, avoids the publication of more new names while safeguarding morphological diagnosability. The species treated here as *Hydrochorea* form a morphologically homogeneous group in terms of vegetative and floral characters, although the fruits are variable as observed in other mimosoid genera (e.g., Aviles Peraza et al. 2022). Nevertheless, fruit type is useful for the identification of some species of *Hydrochorea*. Quantitative characters, such as the number of pinnae per leaf and number of leaflets per pinna, also can be useful for identification of some species, but we commonly observed overlap in these characters between species and even variation on the same individual.

## Supplementary Material

XML Treatment for Hydrochorea

XML Treatment for Hydrochorea corymbosa

XML Treatment for Hydrochorea elegans

XML Treatment for Hydrochorea gonggrijpii

XML Treatment for Hydrochorea leucocalyx

XML Treatment for Hydrochorea marginata

XML Treatment for Hydrochorea obliquifoliolata

XML Treatment for Hydrochorea panurensis

XML Treatment for Hydrochorea pedicellaris

XML Treatment for Hydrochorea rhombifolia

XML Treatment for Hydrochorea uaupensis

## Appendix 1

Vouchers and European Nucleotide Archive (ENA, https://www.ebi.ac.uk/ena/) accession numbers for molecular phylogenetic data. Note that the taxonomy of Albiziasect.Arthrosamanea is updated in this volume by Aviles Peraza et al. (2022), where new binomials in the genus *Pseudalbizzia* Britton & Rose are presented for the majority of the species of this section.

*Acaciarostellifera* Benth., *Murphy 466* (MEL): ERS11697109; *Afrocalliandraredacta* (J.H. Ross) E.R. Souza & L.P. Queiroz, *Germishuizen 5585* (PRE): ERS11697117; *Albiziaadinocephala* Britton & Rose, *Hughes 1070* (FHO): ERS11697120; *Albiziaburkartiana* Barneby & J.W. Grimes, *Stival-Santos 678* (RB): ERS4812854 ; *Albiziacoripatensis* (Rusby) Schery, *Hughes 2433* (FHO): ERS11697123; *Albiziadecandra* (Ducke) Barneby & J.W. Grimes, *Vilhena 231* (NY): ERS11697124; *Albiziaedwallii* (Hoehne) Barneby & J.W. Grimes, *Dalmaso 272* (RB): ERS4812856 ; *Albiziaglabripetala* (H.S. Irwin) G.P. Lewis & P.E. Owen, *Lewis 1652* (K): ERS11697125; *Albiziainundata* (Mart.) Barneby & J.W. Grimes, *Wood 26530* (K): ERS4812859; *Albiziamultiflora* (Kunth) Barneby & J.W. Grimes, *Hughes 3090* (FHO): ERS11697127; *Albizianiopoides* (Spruce ex Benth.) Burkart, *Simon 1601* (CEN): ERS11697128; *Albiziasinaloensis* Britton & Rose, *Hughes 1576* (K): ERS11697130; Albiziasubdimidiatavar.minor Barneby & J.W. Grimes, *Gorts 341* (K): ERS11697131; Albiziasubdimidiata(Splitg.)Barneby & J.W. Grimesvar.subdimidiata, *Ferreira 210* (K): ERS11697129; *Albiziatomentella* Miq., *Leach & Dunlop 3801* (L): ERR9867596; *Albiziatomentosa* Standl., *Hughes 648* (K): ERS11697132; *Albiziaxerophytica* J.Linares, *Hughes 1435* (K): ERS11697133; *Archidendronclypearia* (Jack) I.C. Nielsen, *Wieringa 1849* (WAG): ERS11697136; *Calliandrabella* Benth., *Queiroz 15696* (HUEFS): ERS11697156; *Enterolobiumcyclocarpum* (Jacq.) Griseb., *Macqueen & Styles 75* (K): ERS11697204; *Hydrochoreacorymbosa* (Rich.) Barneby & J.W. Grimes (1), *Bonadeu 655* (RB): ERS4812902 ; *Hydrochoreacorymbosa* (Rich.) Barneby & J.W. Grimes (2), *Iganci 862* (RB): ERS4812903 ; *Hydrochoreaelegans* (Ducke) Iganci, M.V.B. Soares & M.P. Morim, *Iganci 870* (RB): ERS11697146; *Hydrochoreagonggrijpii* (Kleinh.) Barneby & J.W. Grimes, *Tillett 45696* (K): ERS11697223; *Hydrochorealeucocalyx* (Britton & Rose) Iganci, M.V.B. Soares & M.P. Morim, *Aguilar 1939* (NY): ERS11697147; *Hydrochoreapanurensis* (Spruce ex Benth.) M.V.B. Soares, M.P. Morim & Iganci, *Morim 563* (NY): ERS11697224; *Hydrochoreaobliquifoliolata* (De Wild.) E.J.M. Koenen, *Wieringa 6519* (WAG): ERS4812863 ; *Hydrochoreapedicellaris* (DC.) Iganci, M.V.B. Soares & M.P. Morim, *Queiroz 15529* (HUEFS): ERS4812877 ; *Hydrochorearhombifolia* (Benth.) E.J.M. Koenen, *Deighton 3618* (K): ERS11697168; *Hydrochoreauaupensis* M.P. Morim, Iganci & E.J.M. Koenen, *Morim 577* (RB): ERS4812878 ; *Jupunbaabbottii* Britton & Rose, *Zanoni 21220* (NY): ERS11697071; *Jupunbaasplenifolia* Britton & Rose, *Ekman 6383* (NY): ERS11697072; *Jupunbabarbouriana* (Standl.) M.V.B. Soares, M.P. Morim & Iganci, *Iganci 847* (RB): ERS11697073; *Jupunbabrachystachya* (DC.) M.V.B. Soares, M.P. Morim & Iganci, *Lima 7438* (RB): ERS11697074; *Jupunbacochleata* (Willd.) M.V.B. Soares, M.P. Morim & Iganci, *Bonadeu 673* (RB): ERS11697076; *Jupunbacommutata* (Barneby & J.W. Grimes) M.V.B. Soares, M.P. Morim & Iganci, *Maguire 46145* (NY): ERS11697077; *Jupunbafilamentosa*, (Benth.) M.V.B. Soares, M.P. Morim & Iganci *Lima 7487* (RB): ERS11697079; *Jupunbafloribunda* (Spruce ex Benth.) M.V.B. Soares, M.P. Morim & Iganci, *Iganci 883* (RB): ERS11697080; *Jupunbaidiopoda* (S.F. Blake) M.V.B. Soares, M.P. Morim & Iganci, *Quesada 1718* (NY): ERS11697081; *Jupunbalaeta* (Benth.) M.V.B. Soares, M.P. Morim & Iganci, *Mori 25147* (NY): ERS11697083; *Jupunbalangsdorfii* (Benth.) M.V.B. Soares, M.P. Morim & Iganci, *Ribeiro 728* (RB): ERS11697084; *Jupunbaleucophylla* (Spruce ex Benth.) M.V.B. Soares, M.P. Morim & Iganci, *Iganci 839* (RB): ERS11697086; *Jupunbalongipedunculata* (H.S. Irwin) M.V.B. Soares, M.P. Morim & Iganci, *Cardona 2682* (NY): ERS11697088; *Jupunbamacradenia* (Pittier) M.V.B. Soares, M.P. Morim & Iganci, *Lourteig 3021* (NY): ERS11697089; *Jupunbamicrocalyx* (Spruce ex Benth.) M.V.B. Soares, M.P. Morim & Iganci, *Iganci 855* (RB): ERS11697090; *Jupunbanipensis* Britton & Rose, *Mayo 19662* (NY): ERS11697091; *Jupunbaoppositifolia* Britton & Rose, *Liegier 16014* (NY): ERS11697092; *Jupunbaoxyphyllidia* (Barneby & J.W. Grimes) M.V.B. Soares, M.P. Morim & Iganci, *Yonker 6157* (NY): ERS11697093; *Jupunbarhombea* (Benth.) M.V.B. Soares, M.P. Morim & Iganci, *Iganci 261* (RB): ERS11697087; *Jupunbatrapezifolia* Moldenke, *Simon 1600* (CEN): ERS4812839 ; *Jupunbavillosa* (Iganci & M.P. Lima) M.V.B. Soares, M.P. Morim & Iganci, *Borges 423* (RB): ERS11697095; *Punjubacallejasii* (Barneby & J.W. Grimes) M.V.B. Soares, M.P. Morim & Iganci, *Daly 5935* (NY): ERS11697075; *Punjubakillipii* Britton & Rose, *Palodorios 6252* (NY): ERS11697082; *Punjubalehmannii* Britton & Rose ex Britton & Killip, *Escobar 7465* (NY): ERS11697085; *Punjubaracemiflora* Britton & Rose, *Jimenez & Soares 3626* (USJ): ERS11697094.

## Supplementary materials

Supplementary material 1 Supplementary data file S1 Data type zip. archiv. Explanation note Alignments of 560 nuclear loci in FASTA format (zipped). https://binary.pensoft.net/file/733501This dataset is made available under the Open Database License (http://opendatacommons.org/licenses/odbl/1.0/). The Open Database License (ODbL) is a license agreement intended to allow users to freely share, modify, and use this Dataset while maintaining this same freedom for others, provided that the original source and author(s) are credited.Marcos Vinicius Batista Soares, Erik Jozef Mathieu Koenen3, João Ricardo Vieira Iganci, Marli Pires Morim

Supplementary material 2 Supplementary data file S2 Data type zip. archiv. Explanation note Gene trees of 560 nuclear loci in Newick format (zipped). https://binary.pensoft.net/file/733502This dataset is made available under the Open Database License (http://opendatacommons.org/licenses/odbl/1.0/). The Open Database License (ODbL) is a license agreement intended to allow users to freely share, modify, and use this Dataset while maintaining this same freedom for others, provided that the original source and author(s) are credited.Marcos Vinicius Batista Soares, Erik Jozef Mathieu Koenen3, João Ricardo Vieira Iganci, Marli Pires Morim

Supplementary material 3 Supplementary data file S3 Data type zip. archiv. Explanation note MI gene trees (one tip per accession) of 398 nuclear loci with nodes with < 10% bootstrap support collapsed, in Newick format. https://binary.pensoft.net/file/733503This dataset is made available under the Open Database License (http://opendatacommons.org/licenses/odbl/1.0/). The Open Database License (ODbL) is a license agreement intended to allow users to freely share, modify, and use this Dataset while maintaining this same freedom for others, provided that the original source and author(s) are credited.Marcos Vinicius Batista Soares, Erik Jozef Mathieu Koenen3, João Ricardo Vieira Iganci, Marli Pires Morim
